# *TOP1α*, *UPF1*, and *TTG2* regulate seed size in a parental dosage–dependent manner

**DOI:** 10.1371/journal.pbio.3000930

**Published:** 2020-11-06

**Authors:** Chengxiang Li, Ximing Gong, Bin Zhang, Zhe Liang, Chui Eng Wong, Benjamin Yen How See, Hao Yu

**Affiliations:** 1 Department of Biological Sciences, Faculty of Science, National University of Singapore, Singapore; 2 Temasek Life Sciences Laboratory, National University of Singapore, Singapore; University of California Riverside, UNITED STATES

## Abstract

Cues of maternal and paternal origins interact to control seed development, and the underlying molecular mechanisms are still far from clear. Here, we show that *TOPOISOMERASE Iα* (*TOP1α*), *UP-FRAMESHIFT SUPPRESSOR 1* (*UPF1*), and *TRANSPARENT TESTA GLABRA2* (*TTG2*) gametophytically, biparentally regulate seed size in *Arabidopsis*. *TOP1α* and *UPF1* are mainly expressed in antipodal cells, and loss of their function leads to ectopic *TTG2* expression in these female gametophytic cells. We further demonstrate that TOP1α and UPF1 directly repress *TTG2* expression through affecting its chromatin status and determine its relative expression in antipodal cells versus sperm cells, which controls seed size in a dosage-dependent and parent-of-origin-dependent manner. The molecular interplay among these three genes explains their biparental gametophytic effect during diploidy and interploidy reciprocal crosses. Taken together, our findings reveal a molecular framework of parental interaction for seed size control.

## Abstract 2

Cues of maternal and paternal origin interact to control seed development, and the underlying molecular mechanisms are still far from clear. This study shows that in *Arabidopsis*, the relative dosage of the transcription factor TTG2 between antipodal cells and sperm cells at the beginning of seed development determines seed size under the control of TOP1α and UPF1.

## Introduction

The seed of angiosperm arises from double fertilization, in which two sperm nuclei (1n) fuse with the egg cell (1n) and the central cell (2n), respectively. The fertilization product from the sperm-egg fusion, the zygote (2n), develops into the embryo that could potentially generate the new plant, whereas the product from sperm–central cell fusion gives rise to the endosperm (3n), which provides nutrition to the embryo or seedling development. The female gametophyte of *Arabidopsis thaliana* contains other accessory cells in addition to the gametes, including two synergid cells and three antipodal cells. Synergid cells function in pollen tube guidance before and during fertilization, whereas the role of antipodal cells is largely elusive.

The endosperm of *Arabidopsis* undergoes karyokinesis repeatedly at early stages. Subsequently, the cell wall appears to separate individual nuclei, a process called the endosperm cellularization. The timing of this process determines seed size, although embryo enlargement replaces the space of endosperm to fill the seed cavity afterward. The small-seed mutants of the *IKU*-pathway genes, such as *haiku1* (*iku1*), *iku2*, and *miniseed3* (*mini3*), exhibit early endosperm cellularization [1–3], whereas the gain-of-function mutation of *SHORT HYPOCOTYL UNDER BLUE1* (*SHB1*) delays endosperm cellularization, resulting in large seeds [2, 4, 5]. In addition, endosperm development is also affected by neighboring tissues. For example, maternal sporophytic mutation of *TRANSPARENT TESTA GLABRA2* (*TTG2*) restricts integument elongation and causes precocious endosperm cellularization. *TTG2* genetically interacts with the *IKU*-pathway as *ttg2 iku2* double mutants exhibit stronger seed phenotypes than either of the single mutants [6]. However, how *TTG2* is regulated to control seed development is unclear so far. Besides, seed coat–derived small RNAs can also control endosperm development by regulating the gene expression in the endosperm [7, 8].

In *Arabidopsis*, smaller seeds result from pollinating tetraploids by diploids, which is due to early endosperm cellularization. In contrast, the seeds from pollinating diploids by tetraploids are highly abortive because of late or failed endosperm cellularization [9, 10]. The reciprocal phenotypes of interploidy crosses could be due to altered dosage of maternally expressed genes (MEGs) and paternally expressed genes (PEGs), which are imprinted genes exhibiting uniparental expression in the endosperm.

However, endosperm development could also be affected by dosage-dependent factors in addition to imprinting. For example, sole duplication of the endosperm ploidy by nitrous oxide treatment at the beginning of seed development in maize, from 3n (2 maternal versus 1 paternal) to 6n (4 maternal versus 2 paternal), is sufficient to cause defective endosperms. Although the maternal/paternal ratio within this sort of endosperms per se is not changed by nuclear duplication, the dosage balance is altered between the components in the nascent endosperm nucleus and those inherited from female gametophyte [11, 12]. Therefore, the dosage effect could be expanded outside the endosperm nuclei, which is similar to the functional mode of *TTG2* in affecting endosperm cellularization [6]. Furthermore, female sporophytic mutation of *TTG2* suppresses the interploidy abortion without affecting the parental dosage within endosperm [13]. These observations suggest that parental-inherited factors, no matter whether they function in the endosperm or not, may exert dosage-dependent effects to regulate seed size. Nevertheless, the potential non-imprinting parent-of-origin effects are largely elusive in *Arabidopsis*.

Here, we report that *TOPOISOMERASE Iα* (*TOP1α*; a DNA topoisomerase) and *UP-FRAMESHIFT SUPPRESSOR 1* (*UPF1*; an ATP-dependent RNA helicase) regulate seed size though *TTG2* (a WRKY transcription factor). These non-imprinted genes exert parent-of-origin roles, including a major maternal gametophytic effect and a minor paternal effect. Loss of *TOP1α* or *UPF1* leads to ectopic expression of *TTG2* in antipodal cells. TOP1α and UPF1 directly repress *TTG2* by affecting its chromatin status. Genetic analysis consistently shows that *TOP1α* and *UPF1* function upstream of *TTG2*. Our findings suggest that the relative *TTG2* dosage in antipodal cells to sperms determines seed size, thus revealing a novel parental dosage–sensitive molecular framework that mediates the interplay of maternal and paternal cues in seed development.

## Results

### TOP1α interacts with UPF1 to regulate seed size

**Fig 1:**
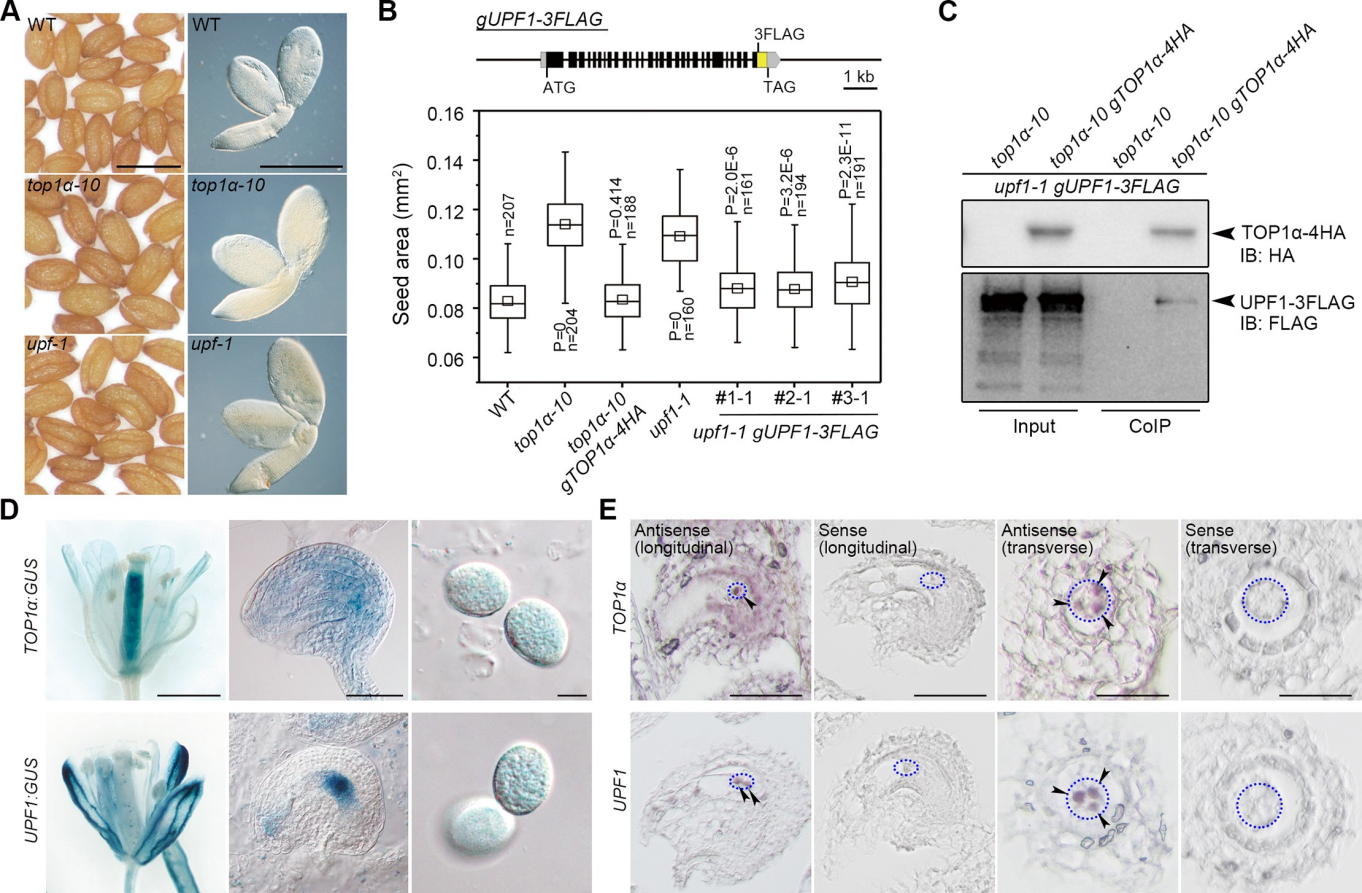
TOP1α and UPF1 regulate seed size and interact in the nucleus. (A) *top1α-10* and *upf1-1* produce larger seeds than WT. Left panels, mature seeds; right panels, mature embryos. Scale bars, 0.5 mm. (B) Seed sizes of *top1α-10* and *upf1-1* are rescued by correspondent genomic fragments. Upper panel: schematic diagram of the *gUPF1-3FLAG* construct. Lower panel: seed sizes of different genetic backgrounds. *P* values were determined by two-tailed Mann-Whitney U-test compared to WT. (C) In vivo interaction between TOP1α and UPF1 shown by CoIP. Nuclear protein extracts from pistils of the specified genotypes were immunoprecipitated by anti-HA antibody. The input and CoIP proteins were detected by anti-HA (upper panel) and anti-FLAG antibody (lower panel), respectively. (D) Representative GUS staining of *TOP1α*:*GUS* and *UPF1*:*GUS* in opening flowers (left panels), ovules (middle panels), and pollen grains (right panels). Scale bars, 1 mm, 50 μm, 10 μm from left to right. (E) In situ localization of *TOP1α* and *UPF1* mRNA expression in ovules. Blue dotted ellipses indicate the regions where antipodal cells are located, and arrowheads indicate antipodal cells with signals. Scale bars, 50 μm, 50 μm, 25 μm, and 25 μm from left to right. The data underlying this figure are included in S1 Data and S1 Raw Images. CoIP, co-immunoprecipitation; GUS, β-glucuronidase; HA, hemagglutinin; n, number of seeds examined; IB, immunoblotting; WT, wild type.

Topoisomerases add or remove DNA supercoils accumulated during replication and transcription [14]. In *Arabidopsis*, *TOP1α* (*AT5G55300*) is the major type I topoisomerase [15]. Interestingly, *TOP1α* not only affects flowering time [16] but also influences seed size (Fig 1A and 1B and S1A Fig). To identify interacting partners of TOP1α, we performed co-immunoprecipitation (CoIP) coupled with liquid chromatography coupled with tandem mass spectrometry (LC-MS/MS) using nuclear extracts of young siliques from the established *top1α-10 gTOP1α-4HA* line [16]. A peptide corresponding to UPF1 was identified, and the corresponding loss-of-function mutant *upf1-1* also produced large seeds (Fig 1A and 1B and S1A–S1C Fig) as reported previously [17]. Notably, the large-seed phenotype of *top1α-10* and *upf1-1* was not due to low fertilization in siliques (S1D Fig). The genomic fragment of *TOP1α* or *UPF1* restored the seed size in the respective mutants, indicating that they are essential for regulating seed size (Fig 1B). Their interaction in vivo was subsequently confirmed by CoIP using the extracts from the homozygous progenies created from *top1α-10 gTOP1α-4HA* crossed with *upf1-1 gUPF1-3FLAG* (Fig 1C).

### TOP1α and UPF1 share an overlapping expression pattern

Both *TOP1α* and *UPF1* were highly expressed in flowers and nascent siliques, but their expression decreased during seed development (S1E and S1F Fig). We generated β-glucuronidase (GUS) reporter lines to visualize their expression in detail. *TOP1α*:*GUS* staining signal appeared in the pistil and the whole ovule with a relatively strong signal at the chalazal end of embryo sac, whereas *UPF1*:*GUS* signal was observed as dots in the pistil and at the chalazal end (Fig 1D). Both *TOP1α*:*GUS* and *UPF1*:*GUS* were weakly expressed in pollen grains (Fig 1D), which is consistent with pollen transcriptome data [18]. To confirm the expression of *TOP1α* and *UPF1* at the chalazal end, we performed in situ hybridization and found that *UPF1* was specifically localized in the three antipodal cells, whereas *TOP1α* was expressed in antipodal cells and their surrounding region (Fig 1E).

Although cytosolic UPF1 is involved in non-sense mRNA decay (NMD) together with UPF2 and UPF3 [19], loss-of-function of UPF2 and UPF3 did not produce large seeds as *upf1-1* (S1G Fig), implying a non-NMD function of UPF1. Although UPF1 was mostly localized in the cytosol, but also present in the nucleus, its interaction with TOP1α was only detected in the nucleus in bimolecular fluorescence complementation (BiFC) assay (S1H–S1J Fig). Moreover, UPF1-3FLAG was co-immunoprecipitated with histone H3, supporting its chromatin association in vivo (S1K Fig). We also inserted a nuclear localization signal (NLS) at the proximal C-terminal of UPF1 to generate *gUPF1-NLS-3FLAG*. The derived fusion protein, which was only localized in the nucleus in planta (S1L Fig), fully rescued the seed phenotype of *upf1-1* (S1M Fig), demonstrating that nuclear-localized UPF1 regulates seed size.

### *top1α-10*, *upf1-1*, and *ttg2-6* exhibit a parent-of-origin effect on diploidy crosses

**Fig 2:**
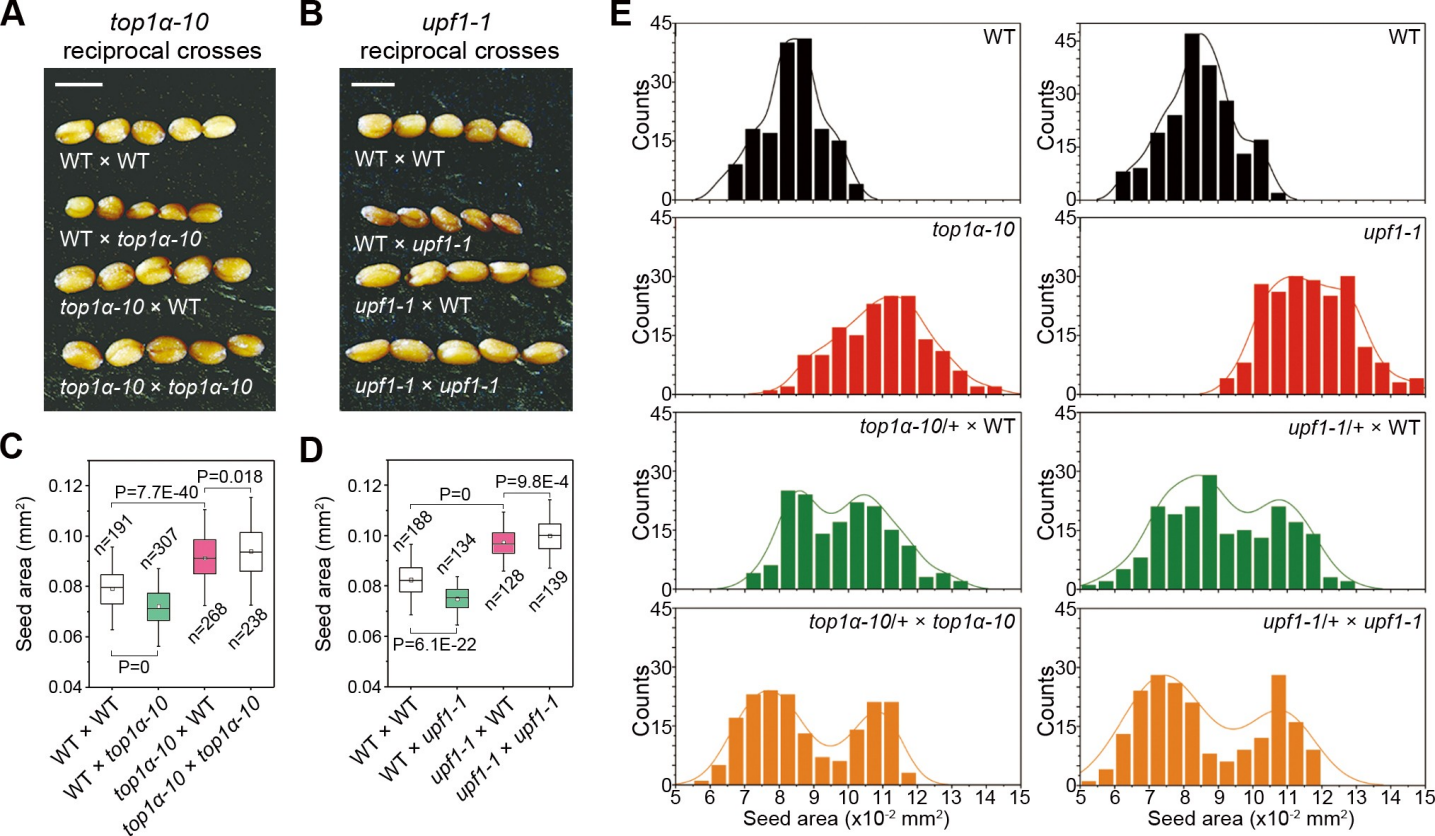
*top1α-10* and *upf1-1* exhibit a parent-of-origin effect on diploidy crosses. (A) Representative seeds from reciprocal crosses between WT and *top1α-10*. Scale bars, 0.5 mm. (B) Representative seeds from reciprocal crosses between WT and *upf1-1*. Scale bars, 0.5 mm. (C, D) Parent-of-origin effect of *top1α-10* (C) and *upf1-1* (D) pertaining to seed size. (E) Histograms of the size distributions of seeds produced by *top1α-10*-related testcrosses (left panels) and *upf1-1-*related testcrosses (left panels). The distribution curves were determined by kernel-density estimation. The data underlying this figure are included in S1 Data. WT, wild-type plants.

We performed reciprocal crosses between *top1α-10* or *upf1-1* and wild-type plants to test the potential parent-of-origin effect. For *top1α-10* and *upf1-1*, their maternal and paternal mutations generated larger and smaller seeds than wild-type plants, respectively (Fig 2A–2D). On the one hand, seeds of hand-pollinated *top1α-10* and *upf1-1* were of similar size to the seeds of *top1α-10* and *upf1-1* pollinated with wild-type plants, indicating their maternal effects (Fig 2A–2D). There were bimodal distributions of seed size of heterozygotes (*top1α-10/+* or *upf1-1/+*) pollinated with homozygous mutants (*top1α-10* or *upf1-1*) or wild-type plants, which supports the maternal gametophytic effect (Fig 2E). This is in line with their overlapping localization in female gametophyte (antipodal cells; Fig 1E). On the other hand, wild-type plants pollinated with *top1α-10* or *upf1-1* produced smaller seeds than self-crossed wild-type plants, indicating their paternal effect. However, such a paternal effect could be overridden by the dominant effect of a maternal mutation of either *TOP1α* or *UPF1* (Fig 2C and 2D). These data suggest that the seed size is determined by the dosage-dependent maternal and paternal *TOP1α* or *UPF1*.

**Fig 3:**
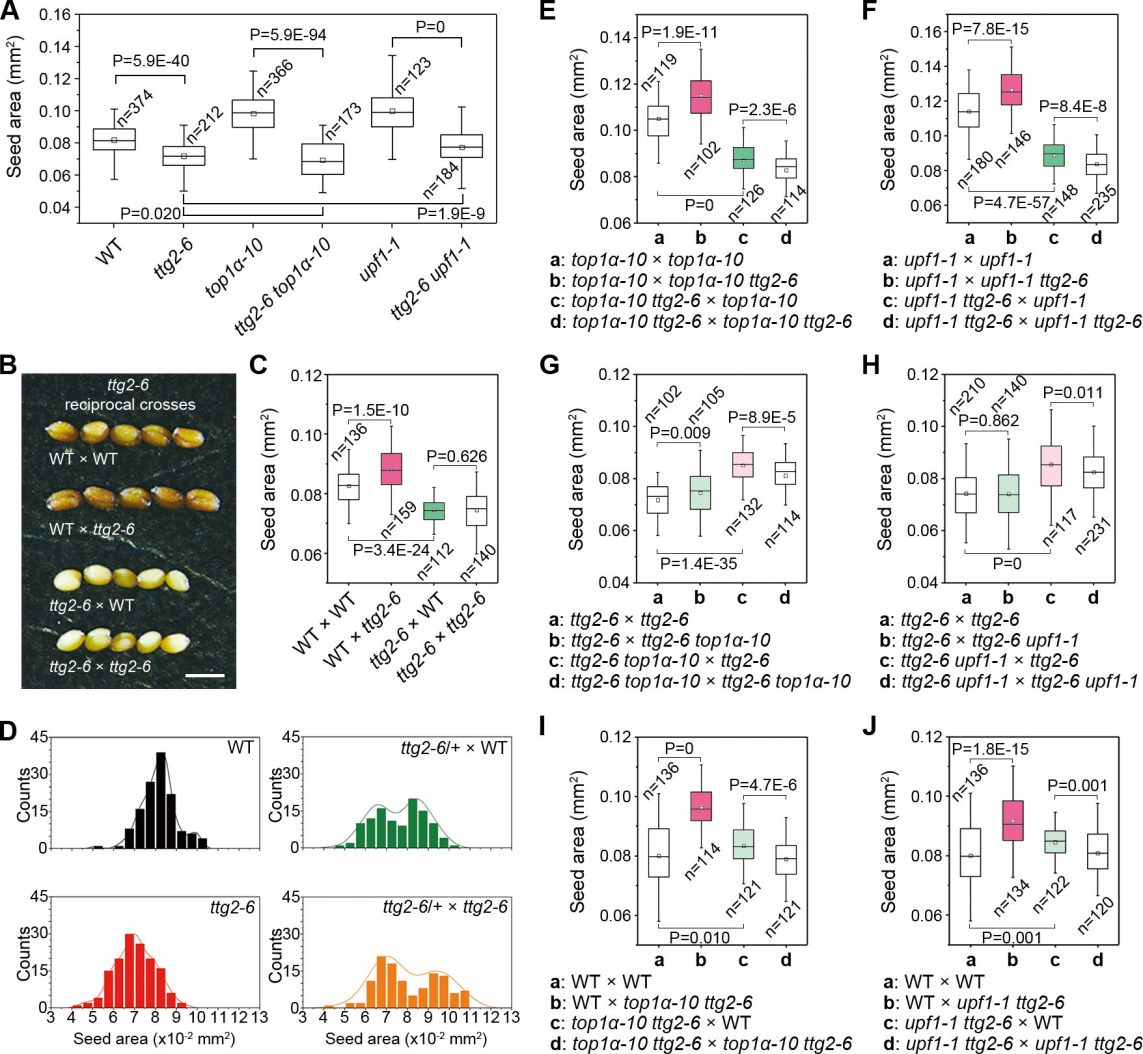
*TTG2* acts downstream of *TOP1α* and *UPF1*. (A) *ttg2-6* is epistatic to *top1α-10* and *upf1-1* pertaining to seed size. (B) Representative seeds from reciprocal crosses between WT and *ttg2-6*. Scale bars, 0.5 mm. (C) Parent-of-origin effect of *ttg2-6* pertaining to seed size. (D) Histograms of the size distributions of seeds produced by *ttg2-6*-related testcrosses. The distribution curves were determined by kernel-density estimation. (E, F) Parent-of-origin effect of *ttg2-6* in *top1α-10* (E) and *upf1-1* backgrounds (F). (G, H) Parent-of-origin effect of *top1α-10* (G) and *upf1-1* (H) in *ttg2-6* background. (I, J) Parent-of-origin effect of *ttg2-6 top1α-10* (I) and *ttg2-6 upf1-1* (J) pertaining to seed size. (A, C, and E-J) *P* values were determined by two-tailed Mann-Whitney U-test. The data underlying this figure are included in S1 Data. n, number of seeds examined; WT, wild-type plants.

The *TTG2* (*AtWRKY44*) has been reported to regulate interploidy barrier as maternal *TTG2* mutations confer tolerance to paternal excess (diploids pollinated with tetraploids) abortion [13], implying a maternal influence on paternal dosage. We then carried out a detailed investigation on *ttg2-6* (SALK_206852) in the Col background (S2A Fig), which produced small seeds like other *ttg2* alleles [20] (Fig 3A). *ttg2-6* was epistatic to both *top1α-10* and *upf1-1* (Fig 3A) and had an opposite gametophytic parent-of-origin effect compared to *top1α-10* and *upf1-1* (Fig 3B–3D). Moreover, the parent-of-origin effect of *ttg2-6* was observed in the *top1α-10* or *upf1-1* background (Fig 3E and 3F), but the same parent-of-origin effect of *top1α-10* or *upf1-1* was attenuated in *ttg2-6* (Fig 3G and 3H). In addition, *ttg2-6 top1α-10* or *ttg2-6 upf1-1* double mutants caused a similar effect to *ttg2-6* in reciprocal crosses (Fig 3I and 3J). Collectively, our results indicate that *TTG2* is one of the genetic downstream targets of *TOP1α* and *UPF1* in the control of seed size.

Since it has been reported that *TTG2* functions as a sporophytic regulator in the integument in L*er* [6] and is also a female gametophytic expressed gene (*DD91*) [21] before fertilization, it is possible that *TTG2* may combine sporophytic and gametophytic effects. Since L*er* encodes a weak *TTG2* [13], the gametophytic role of *TTG2* could be concealed during crosses. Even L*er* × *ttg2-1* resulted in larger seeds than L*er* × L*er*, which cannot be explained by sporophytic maternal effect or integument cell elongation [6]. As *top1α-10* and *upf1-1* did not exhibit seed coat phenotypes like those of *ttg2-6* (S2B Fig), the seed size phenotype of *top1α-10* and *upf1-1* is likely related to the gametophytic rather than the sporophytic *TTG2*.

### TOP1α and UPF1 directly regulate *TTG2*

**Fig 4:**
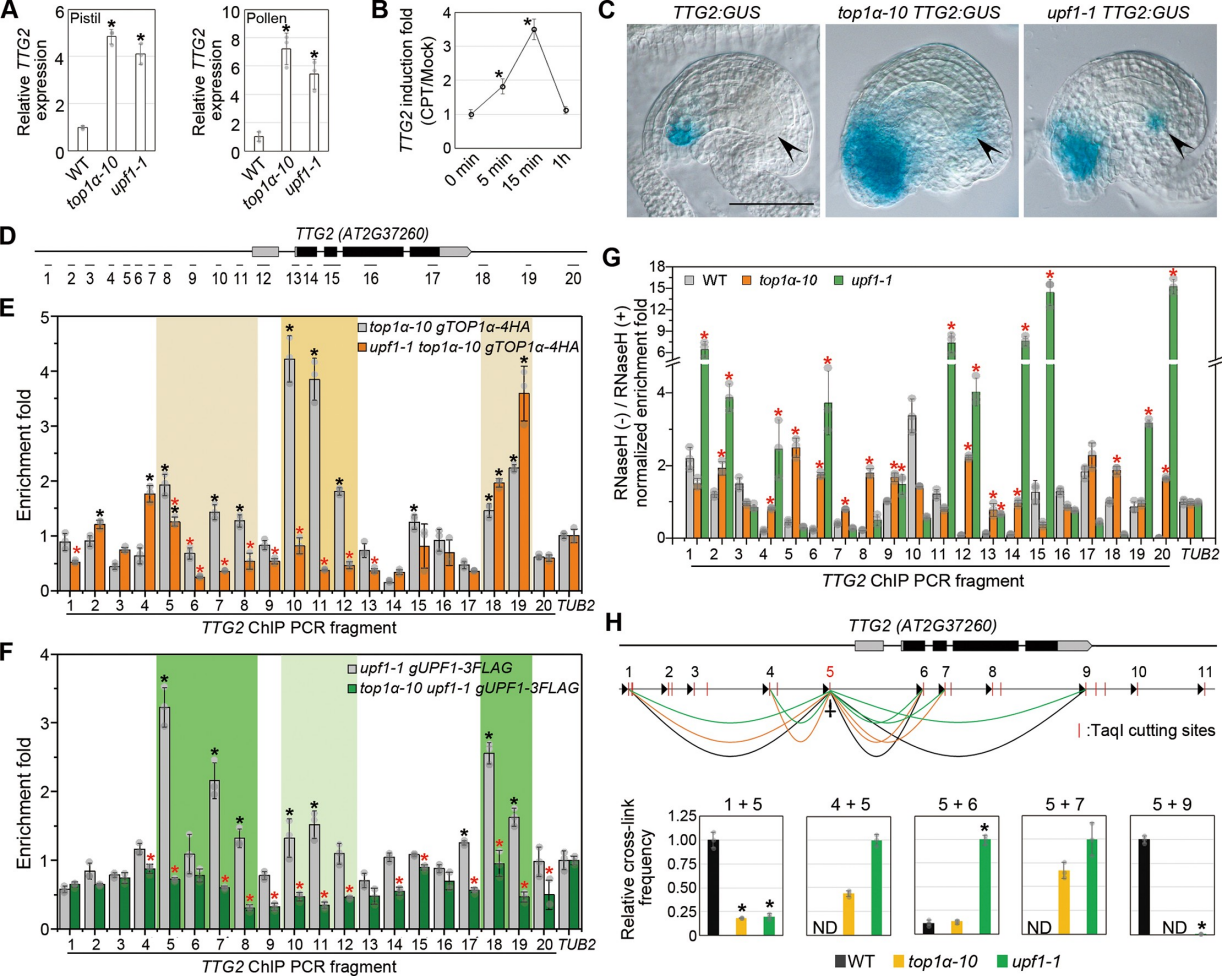
TOP1α and UPF1 directly repress *TTG2*. (A) *TTG2* expression in pistils (left panel) or pollens (right panel) in different genetic backgrounds. Expression values normalized against *U-BOX* are shown relative to the expression level in WT. Values are mean ± s.d. of three biological replicates. *P < 0.05 as compared to WT, two-tailed Student’s *t* test. (B) CPT treatment induces *TTG2* expression. Time-course experiments were conducted by treating the pistils with 10 μM CPT or mock solution. Fold change of CPT-treated versus mock-treated at each time point is presented as mean ± s.d. of three biological replicates. *P < 0.05 as compared to “0 min,” two-tailed Student’s *t* test. (C) Representative GUS staining of *TTG2*:*GUS* in WT (left), *top1α-10* (middle), and *upf1-1* (right) backgrounds. Scale bar, 50 μm. Arrowheads indicate antipodal cells. (D) Schematic diagram of the fragments amplified in ChIP and DRIP analysis spanning the *TTG2* genomic region. The coding and untranslated regions are indicated by black and gray boxes, respectively, and introns and other genomic regions are indicated by black lines. (E, F) ChIP analysis of TOP1α-4HA (E) and UPF1-3FLAG (F) binding to the *TTG2* genomic region. Pistil samples were harvested for ChIP analysis using anti-HA (E) or anti-FLAG (F). Values are mean ± s.d. of three biological replicates. The black asterisks indicate significant enrichment compared to that of *TUB2*. The red asterisks indicate significantly decreased enrichment when UPF1 is absent (E) or TOPα is absent (F). *P < 0.05, two-tailed Student’s *t* test. (G) DRIP analysis at *TTG2* genomic region. Pistil samples were harvested for DRIP analysis using S9.6 antibody. Values are mean ± s.d. of three biological replicates. The red asterisks indicate significantly higher enrichment in comparison with WT. *P < 0.05, two-tailed Student’s *t* test. (H) 3C analysis of chromatin looping at the *TTG2* locus. Upper panel: Schematic diagram of the spatial interactions at *TTG2* genomic locus. Detectable spatial interactions are linked by black (in WT), orange (in *top1α-10*), and green arcs (in *upf1-1*) between the anchor point (site 5) and surrounding loci. Arrowheads indicate the primers for qPCR. Lower panel: the cross-link frequencies are shown relative to the strongest interaction at each site. Values are mean ± s.d. of three biological replicates. *P < 0.05 as compared to WT, two-tailed Student’s *t* test. The data underlying this figure are included in S1 Data. 3C, chromatin conformation capture; ChIP, chromatin immunoprecipitation; CPT, camptothecin; DRIP, DNA:RNA hybrid immunoprecipitation; GUS, β-glucuronidase; HA, hemagglutinin; ND, not detectable; qPCR, quantitative PCR; WT, wild-type plants.

To understand the molecular link among *TOP1α*, *UPF1*, and *TTG2*, we proceeded to investigate their expression profiles. Quantitative real-time PCR revealed that *TTG2* expression was up-regulated in both pistils and pollens of *top1α-10* and *upf1-1* (Fig 4A). Moreover, *TTG2* expression increased during early seed development till 4 days after pollination (DAP) (S2C Fig), whereas the expression of *TOP1α* and *UPF1* decreased (S1F Fig). Camptothecin (CPT; a topoisomerase I–specific inhibitor) treatment also quickly induced *TTG2* expression in pistils (Fig 4B). We generated a transcriptionally fused *TTG2*:*GUS* reporter line, which exhibited the typical trichome signal as previously reported [20] (S2D Fig), suggesting that the reporter line could be used to examine *TTG2* expression. Using this reporter, we observed ectopic signals in antipodal cells and higher signals around the micropyle in *top1α-10* or *upf1-1* compared to wild-type plants (Fig 4C). Ectopic *TTG2* expression in antipodal cells was in line with the localization of *TOP1α* and *UPF1* and persisted at least until 1 DAP (S2E Fig). Despite the known sporophytic *TTG2* effect on seed size [6], our results also revealed a gametophytic effect of *ttg2-6* (Fig 3D), which is consistent with the gametophytic effect exhibited by *top1α-10* and *upf1-1* (Fig 2E). Although *TTG2* expression was also elevated in the whole micropyle region (including the sporophytic tissues) of *top1α-10* and *upf1-1*, these two mutants did not show any seed coat phenotypes related to *ttg2-6* (S2B Fig). These observations indicate that ectopic expression of *TTG2* in antipodal cells rather than in micropyle contributes to the gametophytic effect of *ttg2-6* associated with the seed phenotypes exhibited by *top1α-10* and *upf1-1*.

We further detected the binding of TOP1α and UPF1 to the *TTG2* genomic locus by chromatin immunoprecipitation (ChIP) assay (Fig 4D–4F). The UPF1-NLS-3FLAG displayed stronger binding than UPF1-3FLAG to similar regions (S3A Fig), further supporting the role of nuclear-localized UPF1. Interestingly, there was an overlap in the regions bound by TOP1α and UPF1, although their binding preference differed (Fig 4E and 4F). TOP1α binding to the proximal promoter region was strikingly weakened in *upf1-1*, whereas UPF1’s binding to the whole region was also weakened in *top1α-10* (Fig 4E and 4F), indicating that their protein interaction may strengthen their respective binding to the *TTG2* locus.

Loss of TOP1 could accumulate R-loops (DNA:RNA hybrid) as previously reported [22, 23]. DNA:RNA hybrid immunoprecipitation (DRIP) revealed that R-loops were accumulated in the 5′ *TTG2* promoter region in *top1α-10* and in the entire *TTG2* gnomic region in *upf1-1* (Fig 4G). Higher expression of *TTG2* in *top1α-10* and *upf1-1* suggests a promotional role of the R-loops, as they can keep chromatin open and protect hypomethylated promoters from being silenced [24, 25]. Through chromatin conformation capture (3C) assays of either TaqI- or CviQI-digested chromatin, we found that the proximal promoter region spatially interacted with the distal 5′ and 3′ regions, indicating a folded chromatin in wild-type plants (Fig 4H and S3B Fig). In *top1α-10* and *upf1-1*, the distal chromatin interactions were significantly weakened, whereas the short-range interactions were strengthened (Fig 4H and S3B Fig). Moreover, we conducted formaldehyde-assisted isolation of regulatory elements coupled with quantitative PCR (FAIRE-qPCR) to check the chromatin accessibility. The open chromatin was predominantly located at the coding and distal promoter regions, which became more accessible in *top1α-10* and *upf1-1* (S3C Fig). These data substantiate that loss of TOP1α and UPF1 decondenses the *TTG2* locus.

We also tested if R-loops could protect hypomethylated promoters from being silenced. The *met1* (mainly CpG hypomethylation), but not *cmt3* (mainly CpHpG hypomethylation), showed a parent-of-origin effect on seed size [26]. *ago4 ago6*, *nrpd2a-2 nrpd2b-1*, and *cmt3 drm1 drm2* had greatly hypomethylated CpHpH or CpHpG sites [27, 28] but did not display parent-of-origin effects on seed size (S4A–S4C Fig). Thus, we only quantified CpG methylation at the *TTG2* locus. Hypomethylated *TTG2* promoters in *top1α-10* and *upf1-1* compared to wild type were revealed by CpG-sensitive restriction enzymes digestion coupled qPCR (S4D and S4E Fig). The hypomethylation was verified by methyl-cytosine immunoprecipitation (mCIP) at the TOP1α-occupied promoter region (m10 and m11) (S4D and S4F Fig). Taken together, our results suggest that TOP1α and UPF1 bind to the *TTG2* locus interdependently and keep this locus folded and silenced. Loss of TOP1α and UPF1 generates ectopic R-loops in this region, resulting in decondensed and hypomethylated chromatin and thus permitting a basal *TTG2* transcription.

### *TOP1α*, *UPF1*, and *TTG2* are non-imprinted genes and provide feedback regulation of ploidy increase

We are wondering how *TTG2*, *TOP1α*, and *UPF1* could contribute to the parent-of-origin effect. Imprinting is the best-studied cause for parent-of-origin effects. However, reciprocal crosses carried out in this study revealed that these genes did not act like either MEG or PEG (S5A and S5B Fig). We found that *TTG2* was biparentally expressed (S5C Fig), and *TTG2*, *TOP1α*, and *UPF1* were also reported as non-imprinted genes [29]. Imprinting happens in the endosperm [30, 31], whereas *TOP1α* and *UPF1* are mainly expressed in antipodal cells rather than central cells (Fig 1D and 1E).

The parent-of-origin effects of *TOP1α*, *UPF1*, and *TTG2*, which are independent of imprinting, imply that they may be involved in a hitherto unknown module that senses and regulates parental dosage balance. Pollinating 2x Col with *top1α-10* produced small seeds (Fig 2A and 2C), which is similar to tetraploids pollinated with diploids. Thus, *top1α-10* likely simulates a decreased genome dosage compared to 2x Col. Interestingly, tetraploids have larger seeds than diploids, whereas *top1α-10* produced large seeds even when it simulates a decreased genomic dosage. Thus, it is highly possible that *TOP1α*, *UPF1*, and *TTG2* act in a feedback loop that compensates for the effects of ploidy increase (S6B Fig). To test this assumption, we generated tetraploid mutants (S7 Fig). *TOP1α* and *UPF1* expression was reduced in the tetraploids versus diploidy wild-type plants, whereas *TTG2* expression was elevated (S6C Fig), indicating that they respond to ploidy increase. Moreover, the seeds of 4x Col were 34.0% larger than those of diploidy wild type. Such an increase weakened in *top1α-10* and *upf1-1* backgrounds (25.9% and 19.1% larger than diploids, respectively) but enhanced in the *ttg2-6* background (48.3% larger than diploids) (S6D Fig). Seeds of 8x *ttg2-6* were larger than those of 4x *ttg2-6*, whereas 8x *top1α-10* produced seeds with similar size to those of 4x *top1α-10* (S6D Fig). These data support that loss of *TTG2* or *TOP1α* enhances or weakens the effect of ploidy increase, respectively. In this scenario, changes in the expression of *TOP1α*, *UPF1*, and *TTG2* may mimic altered parental dosage, resulting in parent-of-origin effect in reciprocal crosses.

### *top1α-10*, *upf1-1*, and *ttg2-6* affect the reciprocal phenotypes of interploidy crosses

**Fig 5:**
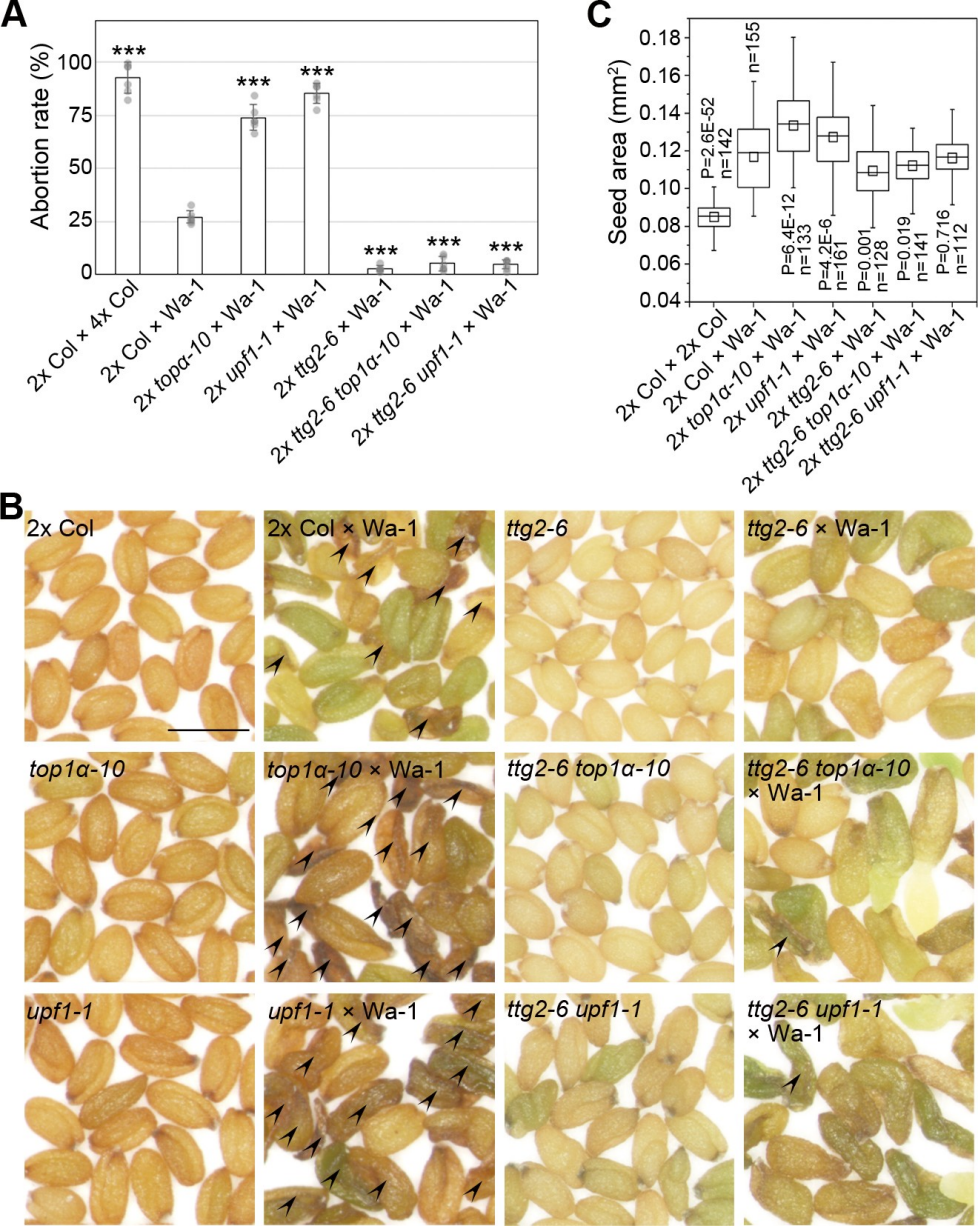
*TOP1α*, *UPF1*, and *TTG2* affect the phenotypes of paternal-excess interploidy crosses. (A) Maternal mutations affect the abortion rate of paternal excess. Values are mean ± s.d. ***P < 0.001 as compared to 2x Col × Wa-1, two-tailed Student’s *t* test. (B) Seed morphology of paternal excess using Wa-1 and diploidy mutants. Arrowheads indicate the aborted seeds. Scale bar, 0.5 mm. (C) Maternal mutations affect the seed size of paternal excess. Aborted seeds are excluded before measurements. *P* values were determined by two-tailed Mann-Whitney U-test as compared to 2x Col × Wa-1. The data underlying this figure are included in S1 Data. n, number of seeds examined; Wa-1, Warschau.

Interploidy crosses are an ideal platform to test the parent-of-origin effect and dosage effect because of the distinct phenotype of paternal excess (diploids pollinated with tetraploids) and maternal excess (tetraploids pollinated with diploids). Moreover, maternal mutation of *TTG2* suppresses the interploidy barrier [13]. Thus, we investigated the effects of *top1α-10*, *upf1-1*, and *ttg2-6* on interploidy crosses as well. Firstly, we examined various mutants in paternal excess. Warschau (Wa-1) is a natural autotetraploid ecotype. Since the abortion rate of 2x Col pollinated with 4x Col or Wa-1 was 92.38% or 27.45%, respectively, Wa-1 was used as a male parent because the interploidy barrier in Col background was too strong to show the potential variances (Fig 5A). Seeds of 2x *top1α-10* or 2x *upf1-1* pollinated with Wa-1 had higher abortion rates than the seeds of 2x Col pollinated with Wa-1, whereas the seeds of 2x *ttg2-6*, 2x *ttg2-6 top1α-10*, or 2x *ttg2-6 upf1-1* pollinated with Wa-1 had lower abortion rates than seeds of 2x Col pollinated with Wa-1 (Fig 5A and 5B). Similarly, maternal *TOP1α* and *UPF1* mutations also strengthened the large-seed phenotype of paternal excess (Fig 5C). In contrast, the seeds of 2x Col pollinated with 4x *top1α-10* or 4x *upf1-1* had lower abortion rates than the seeds of 2x Col pollinated with 4x Col or 4x *ttg2-6* (S8A Fig). Since most of the seeds resulting from these paternal excesses in the Col background were aborted or in irregular shapes (S8B Fig), we did not measure their seed size.

**Fig 6:**
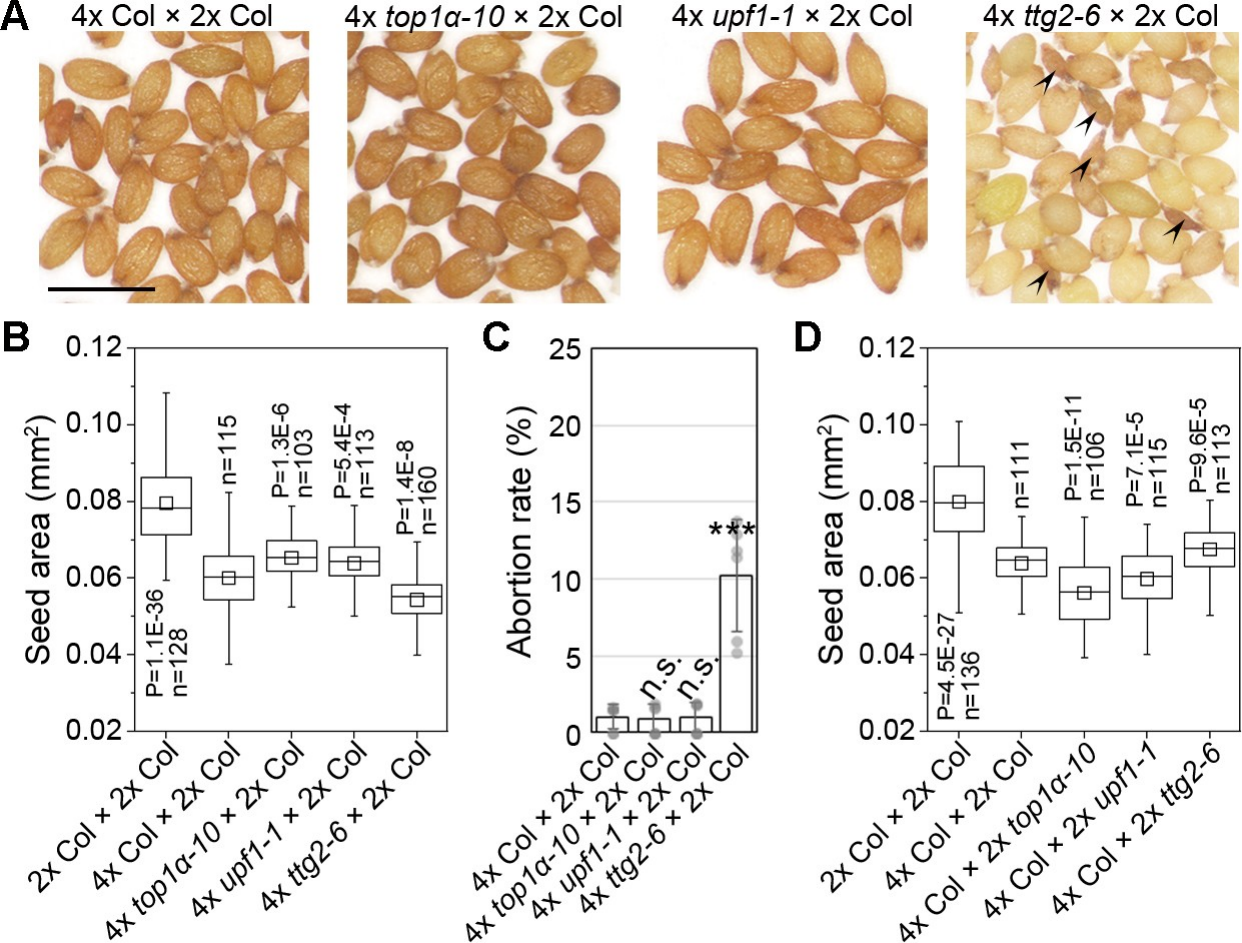
*TOP1α*, *UPF1*, and *TTG2* affect the phenotypes of maternal-excess interploidy crosses. (A) Morphology of the seeds produced by 4x mutants pollinated with diploidy wild type. Arrowheads indicate the aborted seeds. Scale bar, 0.5 mm. (B) Maternal mutations affect the seed size of maternal excess. *P* values were determined by two-tailed Mann-Whitney U-test as compared to 4x Col × 2x Col. (C) Maternal mutations affect the abortion rate of maternal excess. Values are mean ± s.d. ***P < 0.001 as compared to 4x Col × 2x Col, two-tailed Student’s *t* test. (D) Paternal mutations affect the seed size of maternal excess. *P* values were determined by two-tailed Mann-Whitney U-test as compared to 4x Col × 2x Col. The data underlying this figure are included in S1 Data. n, number of seeds examined.

We further investigated seeds produced from maternal excess. Maternal excess produces viable seeds so we can test seed size in the Col background. Pollinating 4x *top1α-10* or 4x *upf1-1* with 2x Col produced larger seeds than pollinating 4x Col with 2x Col, whereas 4x *ttg2-6* pollinated with 2x Col produced smaller seeds than 4x Col pollinated with 2x Col (Fig 6A and 6B). In addition, only 4x *ttg2-6* pollinated with 2x Col produced some small aborted seeds, implying an extreme phenotype of maternal excess as shown in hexaploid pollinated with diploid [10] (Fig 6A and 6C). When pollinating 4x Col with different diploidy males, paternal *TTG2* mutations produced larger seeds, whereas paternal *TOP1α* and *UPF1* mutations produced smaller seeds, compared to the seeds of 4x Col pollinated with 2x Col (Fig 6D). Regarding that *top1α-10* and *upf1-1* simulate a decreased genomic dosage, they caused enlarged dosage differences compared to tetraploids. In contrast, *ttg2-6* simulates an increased genomic dosage, thus narrowing the dosage difference between *ttg2-6* and tetraploids. These results support that the mutations of these genes can skew the parental balance to affect the phenotypes of interploidy crosses.

## Discussion

TOP1α and UPF1 are fundamental regulators of DNA and RNA structures, respectively, and have been reported to affect plant development, including the floral transition [16, 32]. In this study, we have revealed that *TOP1α* and *UPF1* regulate seed size through *TTG2* in a parent-of-origin manner. TOP1α interacts with UPF1, and both of them repress *TTG2* biparentally by maintaining the condensed chromatin at the *TTG2* locus correlated with the level of R-loops and CG-methylation, which is consistent with the observations on the epistatic analysis. This mechanism also brings insight into non-NMD functions of UPF1 as a transcription regulator in *Arabidopsis*, which is implied by other studies in *Drosophila* and human cells [33, 34].

In the developmental scope, seed size is controlled in multiple dimensions, such as the capacity restriction by the integument, the growth of the embryo, and the timing of endosperm cellularization. In addition, parental cues from gametophytes may also contribute to seed development at the very beginning of seed development. For example, pollen-derived *SHORT SUSPENSOR* (*SSP*) mRNA is translated in the zygote to control the asymmetric zygotic cell division [35]. It is noteworthy that *TOP1α* and *UPF1* function gametophytically and are enriched in antipodal cells rather than female gametes, whereas the function of antipodal cells is by now unclear. One hypothesis is that antipodal cells are backup gametes as they can be transformed into gametes as if the normal gamete development failed [36, 37]. Although *TTG2* has been reported to act sporophytically [6], our reciprocal crosses and test crosses reveal a surprising gametophytic role of *TTG2* in seed development. This role is associated with *TTG2* expression in antipodal cells, which is specifically suppressed by *TOP1α* and *UPF1*, and derepression of *TTG2* contributes to the parent-of-origin effect of *top1α-10*, *upf1-1*, and *ttg2-6*. As antipodal cells are not transmitted to the filial generation as gametes, they exist only for a short period of time after fertilization [38], implying that the related regulators may only work in a narrow time window at the beginning of seed development. Besides, as the paternal-inherited mutations of *TOP1α*, *UPF1*, and *TTG2* also plays roles in determining seed size, we believe that the parental interplay related to these genes happens between antipodal cells and sperms.

**Fig 7:**
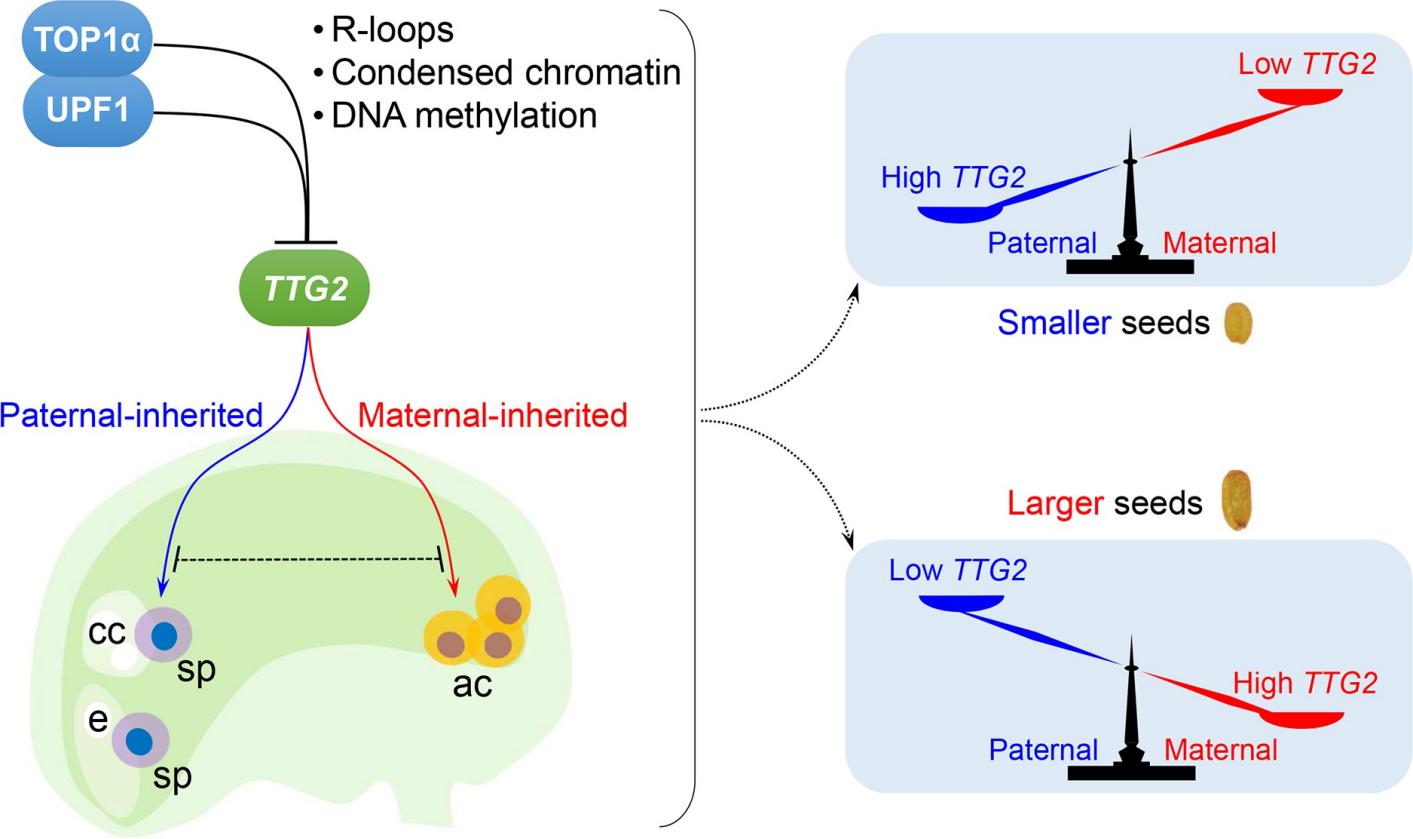
A model depicting the regulation of seed size by parental *TTG2* dosage. TOP1α interacts with UPF1, which facilitates their binding to the *TTG2* locus. This binding compromises R-loops that hamper the chromatin condensation. Loss of *TOP1α* and *UPF1* makes the *TTG2* locus more accessible with less GC-methylation, permitting a higher *TTG2* expression. The repressive effects of TOP1α and UPF1 on *TTG2* exist in sperms from the pollen and in antipodal cells from the female gametophyte, whereas paternal-inherited and maternal-inherited *TTG2* affect seed size in an opposite manner. The relative parental dosage of *TTG2* determines the seed size so that an increase in maternal or paternal *TTG2* results in larger or smaller seeds, respectively. ac, antipodal cells; cc, central cell; e, egg cell; sp, sperm.

Taken together, we propose a *TTG2* dosage–dependent molecular framework wherein the parental *TTG2* dosage between antipodal and sperm cells regulated by *TOP1α* and *UPF1* determines seed size (Fig 7A). Elevated maternal to paternal *TTG2* ratios result in large seeds, whereas the declined ratios cause an opposite phenotype that partially mimics parental imbalance (Fig 7B and S1 Table). This model is consistently valid as evidenced by interploidy crosses. Both *top1α-10* and *upf1-1* simulate a decreased genomic dosage, whereas *ttg2-6* simulates an increased genomic dosage. Thus, they influence the phenotype strength of interploidy crosses. On the other hand, paternal-inherited *top1α-10* or *upf1-1* causes smaller seeds, whereas paternal-inherited *ttg2-6* causes larger seeds in interploidy crosses. In contrast, paternal-inherited *top1α-10*, *upf1-1*, or *ttg2-6* acted opposite to maternal ones. These features are also observed in interploidy crosses, indicating that the parent-of-origin effects of *TOP1α*, *UPF1*, and *TTG2* mutations are not altered by the ploidy level. Therefore, the model described here provides a new layer of regulation on the phenotypes of interploidy crosses. This is reasonable because the molecular framework consisting of *TOP1α*, *UPF1*, and *TTG2* is independent of imprinting, which also affects interploidy phenotypes [39–44].

Our results suggest that a parental dosage–sensitive regulatory module may be controlled by signals existing outside the endosperm in *Arabidopsis*. Although this module acts biparentally, the maternal effect is stronger than the paternal one. For example, the seed phenotypes of homozygous mutants of *TOP1α*, *UPF1*, and *TTG2* are determined by their respective maternal genotype. In addition, maternal mutations of *TOP1α*, *UPF1*, and *TTG2* have a greater influence on the phenotypes of interploidy crosses than their corresponding paternal mutations, respectively. Thus, a key question that remains unanswered is why the maternal and paternal *TTG2* display opposite effects. It is possible that TTG2 protein may have different interacting partners or target genes in antipodal and sperm cells. The antipodal cell fate is controlled by the adjacent gamete, the central cell, so that intensive cell-cell communication should exist between antipodal cells and the central cell [45]. The communication may continuously exist between antipodal cells and the nascent endosperm, which is produced from the fusion between sperm and central cells. Further addressing whether this sort of communication is relevant to different effects of the maternal and paternal *TTG2* may help to understand their downstream regulatory events for seed size control.

## Materials and methods

### Plant materials and growth conditions

*A*. *thaliana* plants were grown under long-day conditions (16 h light/8 h dark) at 22°C. The *top1α-10* (SALK_013164), *upf1-1* (CS6940), *upf2-1* (SAIL_512_G03), *upf3-1* (CS9900), *ttg2-6* (SALK_026852), *cmt3 drm1 drm2* (CS16384), and *nrpd2a-2 nrpd2b-1* (CS66155) are in Col-0 background. The *ago4 ago6* (CS66095) is in L*er* background. The mutants, the natural tetraploid Wa-1 (CS6885) and Bur-0 (CS22679) ecotype, were obtained from ABRC. The 4x Col, 4x *top1α-10*, 4x *upf1-1*, 4x *ttg2-6*, 8x *ttg2-6*, and 8x *top1α-10* were generated in this research by colchicine treatment. Before being used in genetic analysis, the newly generated tetraploids and octoploids were self-pollinated for two generations by single seed descent and checked by flow cytometry in every generation. All transgenic plants were generated by *Agrobacterium tumefaciens*–mediated transformation.

### Ploidy analysis by flow cytometry

One stage 12 flower bud was collected for each plant and was ground to powder in liquid nitrogen in a 1.5-mL tube. Five hundred microliters of TMT buffer (200 mM Tris-HCl [pH 7.5], 4 mM MgCl_2_, 0.5% [v/v] Triton X-100) was added to each sample and vortexed immediately. Fifty microliters of propidium iodide (PI) stain solution (1 mg/mL) was added in each tube and keep at 4°C for 15 min. Filter the slurry by 60-μm membrane before mounting flow cytometry analysis. As the PI staining is sample-amount sensitive, the plant should be grown in the same condition and the flower bud harvested should keep in the same size. Moreover, in each bath of analysis, 2x Col and Wa-1 were involved as controls to calibrate the signals.

### Plasmid construction for plant transformation

The *top1α-10 gTOP1α-4HA* was validated in the previous study [16]. *upf1-1 top1α-10 gTOP1α-4HA* was generated by genetic crossing. To construct *upf1-1 gUPF1-3FLAG*, the genomic region of *UPF1* was amplified into two fragments (−1,636 bp to +8,845 bp, and +8,850 bp to +12,658 bp, numbered relative to start code) and the two fragments were inserted before and after the 3xFLAG tag in pC1305, respectively. To construct *upf1-1 gUPF1-NLS-3FLAG*, an artificial nuclear localization sequence (NLS) was incorporated into *gUPF1-3FLAG* fragment at the beginning of exon 27, between Q1129 and A1130. Both pC1305*-gUPF1-3FLAG* and pC1305*-gUPF1-NLS-3FLAG* were transformed into *upf1-1* background. t*op1α-10 upf1-1 gUPF1-3FLAG* was generated by genetic crossing. To construct *UPF1*:*GUS*, 1,636-bp 5′ upstream sequence (from −1,636 to −1) and 3,809-bp 3′ downstream sequence (from +8,850 to +12,658) of *UPF1* were assembled before and after the GUS coding sequence. The entire fragment was cloned to pCAMBIA1300. To construct *TOP1α*:*GUS*, a 2,593-bp 5′ upstream sequence was cloned to pBI101-GUS. Both *UPF1*:*GUS* and *TOP1α*:*GUS* were transformed into wild-type background. To construct *TTG2*:*GUS*, a 3,459-bp 5′ upstream sequence was cloned to pBI101-GUS. *TTG2*:*GUS* was transformed into *upf1-1* background, and then the typical line was crossed with *top1α-10*. The *TTG2*:*GUS* in wild-type and *top1α-10* backgrounds were segregated from the F2 population. The primers used are listed in S2 Table.

### Seed size measurement

Only the seeds growing at the same time and the same condition were used in the comparison. All the seeds of reciprocal crosses were produced by hand-pollination including the homozygous controls. Mature seeds were spread on a plain white paper. Photos were taken by stereomicroscope (Nikon). The seed area and length/width ratio were analyzed by ImageJ. Box plots were visualized by Origin. Boxes indicated upper quartile to lower quartile, whiskers indicated 1.5 interquartile range (IQR), the means were shown by open squares, and the medians were shown by transverse lines. Two-tailed Mann-Whitney U-tests were applied between samples by Origin, and the *P* values were indicated with the number of the seeds examined.

### Microscopy and histochemical analysis

For the clearing of ovules and developing seeds, the pistils or young siliques were fixed with 10% acetic acid in ethanol for 1 h, washed for 0.5 h in 90% ethanol, 0.5 h in 70% ethanol, and then cleared overnight in chloralhydrate solution (8 g chloralhydrate, 2 mL glycerol, and 4 mL H_2_O). The samples were observed under differential interference contrast (DIC) microscopy (Leica). GUS staining was conducted by normal procedure and the samples were cleared in chloralhydrate solution before being mounted to microscopy.

### In situ hybridization

Unfertilized pistils were cut longitudinally before being fixed in 4% paraformaldehyde at 4°C overnight. They were then dehydrated through an ethanol series and Histoclear, embedded into paraffin, sectioned to 8 μm, and mounted on poly-d-lysine-coated slides (Fisher Scientific). For the synthesis of RNA probes, a gene-specific region of *TOP1α* or *UPF1* was amplified by a pair of primers (S2 Table), cloned into the pGEM-T Easy vector (Promega), and in vitro transcribed using the DIG RNA Labelling Kit (Roche). In situ hybridization was performed as previously reported [46].

### Cell-fraction assay

The cell-fractionation analysis was carried out as described before with modifications [47]. Pistils were ground in liquid nitrogen and were lysed with nuclear fractionation buffer (20 mM Tris-HCl [pH 7.0], 250 mM sucrose, 25% glycerol, 20 mM KCl, 2 mM EDTA, 2.5 mM MgCl_2_, 30 mM β-mercaptoethanol, protease inhibitor cocktail, and 0.7% Triton X-100). The obtained slurry was filtrated (BD Falcon, 100 μM cell strainer) to remove tissue debris, and then the total filtrate was centrifuged at 1,000*g* for 5 min. The supernatant was the cytosolic fraction and record the volume (V^cyt^). The supernatant was filtrated with membrane filters (0.22-μm pore size) to avoid nuclear contamination. The pellet was further washed with resuspension buffer (20 mM Tris-HCl [pH 7.0], 20 mM KCl, 2 mM EDTA, 2.5 mM MgCl_2_, 30 mM β-mercaptoethanol, protease inhibitor cocktail) three to four times until the pellet was no longer green, and the white pellet was resuspended as nuclear fraction in nuclear lysis buffer (50 mM HEPES [pH 7.5], 150 mM NaCl, 1 mM EDTA, 1% SDS, 1% Triton X-100, 30 mM β-mercaptoethanol, protease inhibitor cocktail) and record the volume (V^nuc^). Loadings of fractions were normalized by V^cyt^ and V^nuc^ ratio. Samples were loaded to SDS-PAGE and detected by anti-FLAG antibody (F3165, sigma, 1:5,000 dilution).

### CoIP assay

Pistils in various genetic backgrounds were collected, and the cell fractionation was carried out as described above. Nuclear protein extracts were 4-fold diluted by extraction buffer (50 mM Tris-HCl [pH 7.5], 150 mM NaCl, 1 mM EDTA, 5% glycerol, 0.5% Triton X-100, 1 mM PMSF, proteinase inhibitor cocktail, and 25 μM MG132) and incubated with anti-HA agarose beads (Sigma) or anti-FLAG magnetic beads (Sigma) at 4°C for 2 h and then washed four to six times by extraction buffer. Immunoprecipitated proteins and nuclear protein extracts as inputs were resolved by SDS–polyacrylamide gel electrophoresis and detected by the corresponding antibody (anti-FLAG: F3165, sigma, 1:5,000 dilution; anti-HA: sc-7392 HRP, Santa Cruz, 1:2,000 dilution; anti-H3: ab1791, abcam, 1:3,000 dilution). For IP-MS, immunoprecipitated proteins were eluted and analyzed by TripleTOF 5600 System (AB Sciex).

### Transient expression assays in tobacco

For subcellular localization analysis, coding sequences of *TOP1α* was fused with EGFP at C-terminus in pC1302E, whereas the coding sequence of *UPF1* was fused with mCherry at C-terminus in pC1300mCherry. For BiFC, the coding sequence of *TOP1α* was fused to cYPF at C-terminus in pXY104, whereas the coding sequence of *UPF1* was fused to nYFP at N-terminus in pXY106. The plasmids were transformed into *A*. *tumefaciens*, and then the transformed *A*. *tumefaciens* were infiltrated into *Nicotiana benthamiana* leaves. Leaves were observed 2 d after infiltration under a confocal microscope (Zeiss LSM710).

### ChIP assay

ChIP assays were carried out in various genetic backgrounds using pistils, following the previous protocol with minor modifications [48]. The extracted chromatin was sonicated to produce DNA fragments between 200 and 500 bp. The solubilized chromatin was incubated with anti-HA agarose beads (Sigma) and anti-FLAG magnetic beads (Sigma) for 2 h at 4°C. A genomic fragment of *TUBULIN2* (*TUB2*) was amplified as a control. ChIP assays were repeated with three biological replicates. The primers used are listed in S2 Table.

### DRIP assay

Pistils were ground to fine powder in liquid nitrogen, and DNA was extracted by extraction buffer (10 mM HEPES [pH 7.5], 400 mM sucrose, 25 mM EDTA, 1 mM MgCl_2_, 0.5% SDS, 1 mM PMSF, RNase inhibitor). DNA was recovered by phenol/chloroform extraction and then fragmented into length around 500 bp by pulsing sonication on ice. Normalize the DNA concentration of different samples by nanodrop before subjecting to S9.6 antibody (Kerafast) immunoprecipitation. To generate negative controls, RNaseH (NEB) were added during immunoprecipitation. The immunoprecipitation and following qPCR procedure were the same as ChIP assay. The primers used are listed in S2 Table.

### 3C

The 3C procedure was modified from the previous protocol [49]. Pistils were ground to fine powder in liquid nitrogen and were incubated with cross-link buffer (10 mM HEPES [pH 7.5], 400 mM sucrose, 5 mM MgCl_2_, 1 mM EDTA, 1 mM PMSF, proteinase inhibitor cocktail, 1% [v/v] formaldehyde). Vacuum the mixture for 15 min on ice and stop the cross-link reaction by adding glycine to 125 mM, and then vacuum for an additional 2 min. The mixture was diluted four times by nucleus isolation buffer (15 mM PIPES [pH 6.8], 150 mM sucrose, 5 mM MgCl_2_, 60 mM KCl, 15 mM NaCl, 1 mM CaCl_2_, 1 mM PMSF, proteinase inhibitor cocktail, 0.9% Triton X-100) and was incubated for 15 min at 4°C using rotating wheel. The obtained slurry was filtrated (BD Falcon, 100 mM cell strainer) to remove tissue debris, and then the total filtrate was centrifuged at 1,000*g* for 5 min. Wash the pellet once by resuspension buffer (20 mM Tris-HCl [pH 7.0], 20 mM KCl, 2 mM EDTA, 2.5 mM MgCl_2_, 30 mM β-mercaptoethanol, protease inhibitor cocktail). Then the nuclei were resuspended in the 1X NEB buffer 3.1 containing 0.1% SDS and incubated for 10 min at 65°C. TritonX-100 was added to 1% (v/v) and samples were then digested with CviQI (37°C) and TaqI (55°C) overnight. SDS was added to 1.6% (v/v) and heat 10 min at 65°C in order to inactivate the restriction enzyme. Triton X-100 was added to the final concentration 1% (v/v) and then samples were incubated at 37°C for 0.5 h, mixing occasionally. Then, scale up the total volume to 15 mL and ligate DNA by T4 DNA ligase overnight at room temperature. Subsequently, 200 μg of protease K was added and incubated at 65°C overnight to reverse cross-link and digest the protein. Then DNA was purified by phenol-chloroform extraction and ethanol precipitation. The purified DNA was used as qPCR templates. An 8-kb whole genome sequence of *TTG2* was amplified, digested by CviQI and TaqI, and randomly ligated, as a control template. An amplicon of a *TTG2* genome fragment without CviQI and TaqI cutting site (region 17 in ChIP) was used as the internal control in qPCR. The primers used are listed in S2 Table.

### FAIRE

FAIRE procedure was modified from the previous protocol [50]. Pistils in various genetic backgrounds were used to prepare cross-linked chromatin (for “FAIRE” samples) by the same procedure as ChIP assays. “Un-FAIRE” samples were prepared as the same procedure, just without formaldehyde-mediated cross-linking. The suspended chromatin was extracted by phenol:Chloroform:Isoamyl alcohol (25:24:1) three times and then was ethanol-precipitated before qPCR. Quantification of FAIRE/un-FAIRE ratio was presented to measure chromatin accessibility. The primers used are listed in S2 Table.

### mCIP

Pistils were ground to fine powder in liquid nitrogen. Extract genomic DNA by standard CTAB method. Genomic DNA (250 μg) of each material was sonicated to about 500 bp and subjected to immunoprecipitation by anti-methyl-cytosine. The immunoprecipitation and following qPCR procedure were the same as ChIP assay. The primers used are listed in S2 Table.

### Expression analysis

Total RNA from various tissues was extracted using FavorPrep Plant Total RNA Mini Kit (Favorgen) and reverse-transcribed using the M-MLV Reverse Transcriptase (Promega) according to the manufacturers’ instructions. Quantitative real-time PCR was performed on three biological replicates using the CFX384 real-time PCR detection system with iQ SYBR Green Supermix (Bio-Rad). The expression of *U-BOX* was used as an internal control. Relative expression levels of genes were calculated by the ^Δ^Ct method or ^ΔΔ^Ct method. The primers used are listed in S2 Table.

## Supporting information

S1 Fig TOP1α and nuclear UPF1 regulate seed size. (A) Comparison of 100-seed weight of *top1α-10*, *upf1-1*, and WT. Values are mean ± s.d. Asterisks indicate significant differences in comparison to wild type. *P < 0.05, two-tailed Student’s *t* test. (B) *top1α-10* and *upf1-1* produce slender seeds as indicated by the length/width ratio. *P* values were determined by two-tailed Mann-Whitney U-test compared to WT. (C) The peptide of UPF1 identified by IP-MS/MS. (D) Dissected siliques from plants with various genetic backgrounds. Scale bar, 1 mm. (E) qRT-PCR analysis of the expression profiles of *TOP1α* and *UPF1* in adult plants. Relative expression of GOI was normalized against *U-BOX* expression. Values are mean ± s.d. of three biological replicates. (F) qRT-PCR analysis of the expression profiles of *TOP1α* and *UPF1* during seed development at different days after pollination. Relative expression of GOI was normalized against *U-BOX* expression. Values are mean ± s.d. of three biological replicates. (G) Seed size of NMD-related mutants. The seed size of *upf2-1* is N.A. because of embryonic lethality. *P* values were determined by two-tailed Mann-Whitney U-test in comparison to wild type. (H) Subcellular localization of TOP1α-GFP (upper panels) and UPF1-mCherry (lower panels) in tobacco leaf epidermal cells. Scale bar, 20 μm. (I) BiFC analysis of the interaction between TOP1α and UPF1 in tobacco leaf epidermal cells. Scale bar, 20 μm. (J) UPF1 subcellular localization shown by cell-fractionation assay. UPF1 protein in nuclear (“N”) or cytoplasmic (“C”) fractions extracted from pistils were detected by anti-FLAG. The nuclear fraction was loaded 10-fold in excess compared to the cytosol fraction. The RUBISCO large subunit (RbcL) stained with Ponceau S and immunoblot analysis using anti-H3 are used as the indicators for cytosol and nuclear fractions, respectively. (K) UPF1 is associated with H3 in vivo as revealed by CoIP. Nuclear protein extracts from the pistils were immunoprecipitated by anti-FLAG. The input and co-immunoprecipitated proteins were detected by anti-FLAG and anti-H3. (L) UPF1-NLS subcellular localization shown by cell-fractionation assay. Upper panel, schematic diagram of the *gUPF1-NLS-3FLAG* construct. UPF1-NLS protein in nuclear (“N”) or cytoplasmic (“C”) fractions extracted from pistils were detected by anti-FLAG. The nuclear fractions and cytosolic fractions were loaded in an equivalent dose. (M) The seed size of *upf1-1* is rescued by *gUPF1-NLS-3FLAG*. *P* values were determined by two-tailed Mann-Whitney U-test compared to wild type. The data underlying this figure are included in S2 Data and S1 Raw Images. BiFC, bimolecular fluorescence complementation; CoIP, co-immunoprecipitation; FB, flower bud; GFP, green fluorescent protein; GOI, genes-of-interest; H3, histone 3; IP-MS/MS, liquid chromatography coupled with tandem mass spectrometry; L, leaf; n, number of seeds examined; N.A., not applicable; NLS, nuclear localization signal; NMD, non-sense mRNA decay; OS, old silique (4–7 days after pollination); qRT-PCR, quantitative real-time PCR; R, Root; St, stem; WT, wild-type plants; YS, young silique (0–4 days after pollination).
(TIF)

S2 Fig Characterization of *ttg2-6* and seed coat phenotypes of *ttg2-6*, *top1α-10*, and *upf1-1*. (A) Characterization of *ttg2-6*. Left panel: Schematic diagram of *ttg2-6* insertion site. The coding and untranslated regions are indicated by black and gray boxes, respectively, and introns and other genomic regions are indicated by black lines. Right panel: Relative *TTG2* expression in WT and *ttg2-6*. Values are means ± s.d of three biological replicates. Asterisks indicate significant differences in comparison to wild type. *P < 0.001, two-tailed Student’s *t* test. (B) Seed coat phenotypes of WT, *ttg2-6*, *top1α-10*, and *upf1-1*. Upper panels: SEM of the seed coat. Scale bar, 50 μm. “C” indicates columella, and “PCC” indicates partially collapsed columella. Middle panels: Seed coat mucilage staining with ruthenium red. Scale bar, 200 μm. Bottom panels: Seed color. Scale bar, 0.5 mm. The data underlying this figure are included in S2 Data. (C) qRT-PCR analysis of the expression profiles of *TTG2* during seed development at different DAP. Relative expression of *TTG2* was normalized against *U-BOX* expression. Values are mean ± s.d. of three biological replicates. (D) Typical trichome expression of *TTG2*:*GUS*. (E) Representative GUS staining *of TTG2*:*GUS* in WT (upper row), *top1α-10* (middle row), and *upf1-1* (bottom row) backgrounds, and at 0 DAP (left column), 1 DAP (middle column), and 2 DAP (right column). Scale bars, 50 μm (left column), 50 μm (left column), and 200 μm (right column). Arrowheads indicate antipodal cells. DAP, days after pollination; GUS, β-glucuronidase; qRT-PCR, quantitative real-time PCR; SEM, scanning electron microscopy; WT, wild-type plants.
(TIF)

S3 Fig *TTG2* locus is decondensed in *top1α-10* and *upf1-1*. (A) ChIP analysis of UPF1-NLS-3FLAG binding to the *TTG2* genomic region. ChIP was performed by anti-FLAG. A *TUB2* fragment was amplified as a negative control. Values are mean ± s.d. of three biological replicates. Asterisks indicate significantly high enrichment in comparison to the *TUB2* fragment. *P < 0.05, two-tailed Student’s *t* test. (B) 3C analysis of chromatin looping status at the *TTG2* locus. Upper panel: Schematic diagram of the spatial interactions at *TTG2* genomic locus. Detectable spatial interactions are linked by black (in wild type), orange (in *top1α-10*), and green arcs (in *upf1-1*) between the anchor point (site 6) and surrounding loci. Arrowheads indicate the primers for qPCR. Lower panel: The cross-link frequencies are shown relative to the strongest interaction at each site. Values are mean ± s.d. of three biological replicates. *P < 0.05 as compared to WT, two-tailed Student’s *t* test. (C) FAIRE analysis of chromatin accessibility of *TTG2* locus. Amplicons of cross-linked samples (FAIRE) versus un-cross-linked samples (un-FAIRE) at each site are presented as mean ± s.d. Asterisks indicate significant differences in comparison to wild type. *P < 0.05, two-tailed Student’s *t* test. The data underlying this figure are included in S2 Data. 3C, chromatin conformation capture; ChIP, chromatin immunoprecipitation; FAIRE, formaldehyde-assisted isolation of regulatory elements; ND, not detectable; qPCR, quantitative PCR; WT, wild-type plants.
(TIF)

S4 Fig CpG methylation levels at the *TTG2* genomic locus. (A-C) Reciprocal crosses of *ago4 ago6* (*ago4/6*) (A), *cmt3 drm1 drm2* (*cdd*) (B), and *nrpd2a-2 nrpd2b-1* (*nrpd2a/2b*) (C) with WT plants. *P* values were determined by two-tailed Mann-Whitney U-test. (D) The recognition map of selected CpG-sensitive restriction enzymes at the *TTG2* locus. The cut sites were indicated in blue (HpyCH4IV), orange (BstUI), red (HhaI), and green (HpaII) strings. The regions to be tested in quantitative real-time PCR are marked as m1 to m23 with the color code of restriction enzymes that digest the corresponding region. PCR fragments of ChIP analysis are aligned above the map. (E) Relative methylation levels at the *TTG2* genomic locus. Genomic DNA from pistil was digested by CpG-sensitive restriction enzymes. Undigested genomic DNA was used as an input. The CpG methylation levels were measured by comparing the digested DNA with the input. The relative methylation levels in *top1α-10* and *upf1-1* backgrounds were normalized against those of WT in each region. Values are mean ± s.d. of three biological replicates. Asterisks indicate significantly low methylation in comparison to WT. *P < 0.05, two-tailed paired Student’s *t* test. (F) mCIP analysis of the selected regions of *TTG2*. ChIP PCR fragment 3 and 12 are selected as controls because of no methylation site (fragment 3) or no difference in methylation levels (fragment 12), as indicated in (D and E). ChIP PCR fragment 10 and 11 overlap with region m9–13 as indicated in (D and E). Values are mean ± s.d. of three biological replicates. The asterisks indicate significantly low enrichment compared to WT. *P < 0.05, two-tailed Student’s *t* test. No statistical difference (n.s), *P* > 0.05. The data underlying this figure are included in S2 Data. ChIP, chromatin immunoprecipitation; mCIP, methyl-cytosine immunoprecipitation; n, number of seeds examined; ND, not detected; WT, wild-type plants.
(TIF)

S5 Fig *TOP1α*, *UPF1*, and *TTG2* form a regulatory module that is independent of imprinting. (A and B) The parent-of-origin effect of *top1α-10* and *ttg2-6* is distinct from that of the mutants of imprinted genes. Schematic diagrams represent the patterns of reciprocal crosses that are related to *top1α-10* (A) and *ttg2-6* (B). Left panel: Actual observation in this study. Middle panel: Patterns under PEG assumption. Right panel: Patterns under MEG assumption. *upf1-1* displays a similar behavior to *top1α-10*. As the maternal PEG and the paternal MEG are not expressed, the corresponding genotypes are indicated as “/” in the PEG and MEG assumption, respectively. Seed size is marked as WT-like (“w”), mutant-like (“m”), or additional type (“a”). (C) *TTG2* is not an imprinted gene. Left panel: The SNP in the coding region of *TTG2*^*Bur-0*^ was used to develop the CAPS marker. Restriction enzyme PsiI digests Bur-0 amplicon, but not Col amplicon. Right panel: CAPS test on cDNA derived from the mRNA of F1 siliques at 2 DAP. CAPS, cleaved amplified polymorphic sequences; DAP, days after pollination; MEG, maternally expressed imprinted gene; PEG, paternally expressed imprinted gene; WT, wild-type plants.
(TIF)

S6 Fig *TOP1α*, *UPF1*, and *TTG2* might work in the feedback regulation of polyploidization responses. (A) Schematic diagrams that summarize the behaviors in reciprocal crosses. *top1α-10* (left panel) and *ttg2-6* (middle panel) displayed distinctive phenotypes compared to tetraploids (right panel). *upf1-1* displays a similar phenotype to *top1α-10*. Seed size of reciprocal crosses is marked as “w” (WT-like), “m” (mutant-like) or “a” (additional type). Seed size of interploidy crosses is marked as “Pex” (paternal excess), “4” (tetraploid), “2” (diploid), or “Mex” (maternal excess). (B) *TOP1α*, *UPF1*, and *TTG2* may compose a feedback console in ploidy increase response. Loss of *TOP1α* or *UPF1* mimics a genome-dosage decrease, whereas loss of *TTG2* mimics a genome-dosage increase. (C) Relative gene expression in pistils in tetraploids compared to diploid WT. The relative expression of *TOP1α*, *UPF1*, *TTG2*, and commonly used control genes are presented. Expression values normalized against *U-BOX* are shown relative to the expression levels in diploid WT. Values are mean ± s.d. of three biological replicates. Asterisks indicate significant differences in comparison to diploid WT. *P < 0.05, two-tailed Student’s *t* test. *TOP1α* expression in 4x *top1α-10* and *UPF1* expression in 4x *upf1-1* were not tested (N.A.). (D) Comparison of seed size among diploids, tetraploids, and octoploids in different genetic backgrounds. The percentages of size increase are based on the means of the seed area. *P* values were determined by two-tailed Mann-Whitney U-test in comparison to corresponding diploids. The data underlying this figure are included in S2 Data. n, number of seeds examined; WT, wild-type plants.
(TIF)

S7 Fig Flow cytometry of diploids, tetraploids, and octoploids. Histograms of 5,000 events are shown with the nuclei in different ploidy indicated as 2C, 4C, 8C, and 16C according to DNA contents. Wild-type plants (Col-0) and Wa-1 (an autotetraploidy accession) are used as controls to calibrate the peaks of 2C, 4C, 8C, and 16C nuclei. Pollinating 8x *ttg2-6* with 4x *ttg2-6* generated 6x *ttg2-6*. This is used to show that the peaks are accurate and sensitive enough for determining the ploidy levels. Stage 12 flower buds were used for flow cytometry analysis. Wa-1, Warschau.
(TIF)

S8 Fig Abortion of the seeds produced by pollinating diploid wild-type plants with different tetraploid mutants. (A) Paternal mutations affect the abortion rate of paternal excess. Values are mean ± s.d. *P < 0.05 as compared to 2x Col × 4x Col, two-tailed Student’s *t* test. (B) Morphology of the seeds produced by paternal excess with 4x mutants as male parents. Asterisks indicate viable seeds. Scale bar, 0.5 mm. The data underlying this figure are included in S2 Data.
(TIF)

S1 Table Summary of the parent-of-origin genotypes and the corresponding parental *TTG2* dosages. (PDF)

S2 Table Primers used in this study. (PDF)

S1 Data The data underlying main figures. From Figs 1B, 2C–2E, 3A, 3C–3J, 4A, 4B, 4E–4H, 5A, 5C, and 6B–6D.
(XLSX)

S2 Data The data underlying supporting figures. From S1A, S1B, S1E–S1G, S1M, S2A, S2C, S3A–S3C, S4A–S4C, S4E, S4F, S6C–S6D and S8A Figs.
(XLSX)

S1 Raw Images Raw images underlying figures. From Fig 1C and S1J–S1L Fig.
(PDF)

## Abbreviations

3C: chromatin conformation capture
BiFC: bimolecular fluorescence complementation
ChIP: chromatin immunoprecipitation
CoIP: co-immunoprecipitation
CPT: camptothecin
DAP: days after pollination
DRIP: DNA:RNA hybrid immunoprecipitation
FAIRE: formaldehyde-assisted isolation of regulatory elements
GUS: β-glucuronidase
iku1: *haiku1*
LC-MS/MS: liquid chromatography coupled with tandem mass spectrometry
mCIP: methyl-cytosine immunoprecipitation
MEG: maternally expressed gene
mini3: *miniseed3*
NLS: nuclear localization signal
NMD: non-sense mRNA decay
PEG: paternally expressed gene
qPCR: quantitative PCR
SHB1: *SHORT HYPOCOTYL UNDER BLUE1*
SSP: *SHORT SUSPENSOR*
TOP1α: *TOPOISOMERASE Iα*
TTG2: *TRANSPARENT TESTA GLABRA2*
UPF1: *UP-FRAMESHIFT SUPPRESSOR 1*
Wa-1: Warschau
