# Supplementary figures and images for: TOP1α, UPF1, and TTG2 regulate seed size in a parental dosage–dependent manner
Source: PLoS Biol. 2020 Nov 6;18(11):e3000930. doi: 10.1371/journal.pbio.3000930 (PMC7673560; doi:10.1371/journal.pbio.3000930)

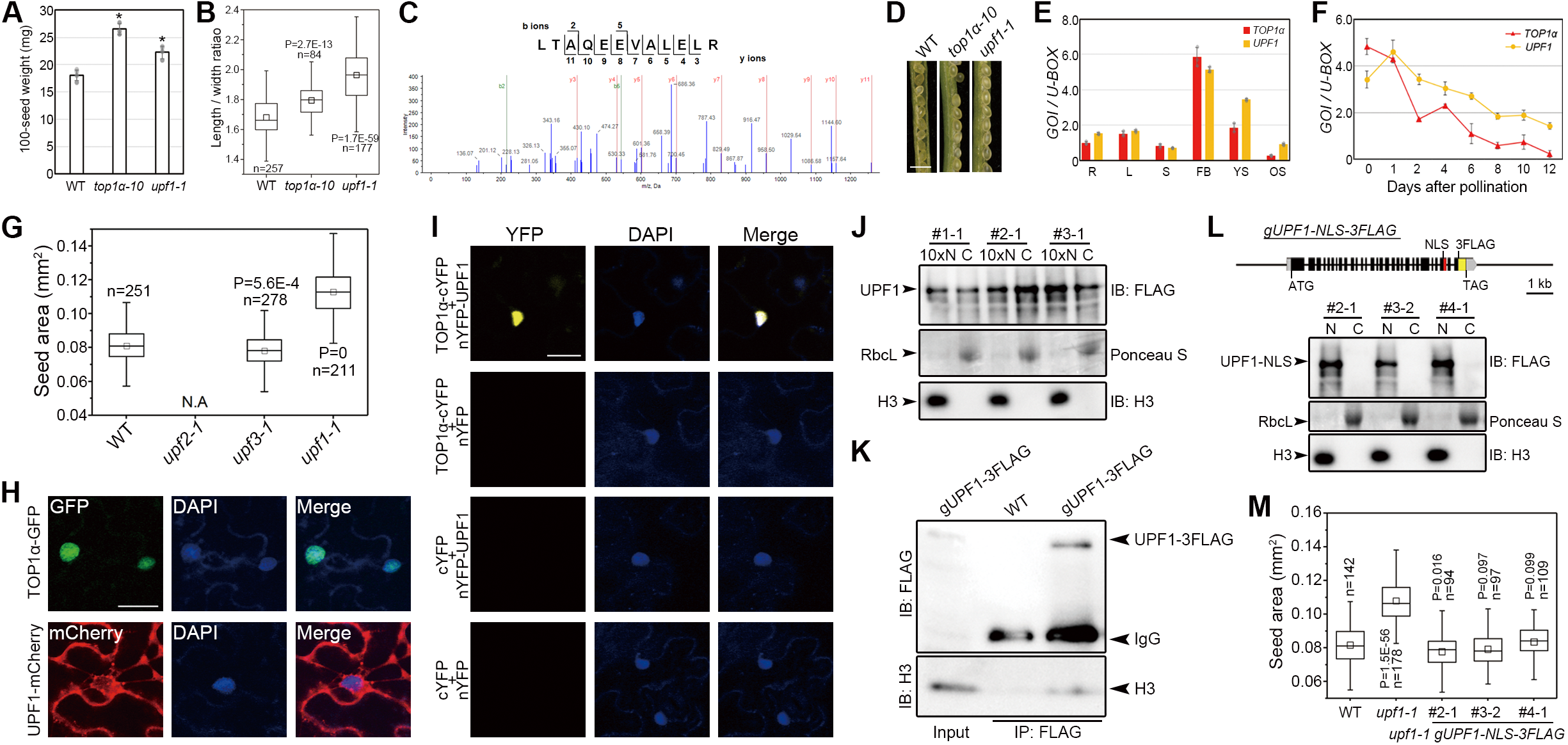

Supplement: S1 Fig — (A) Comparison of 100-seed weight of top1α-10, upf1-1, and WT. Values are mean ± s.d. Asterisks indicate significant differences in comparison to wild type. *P < 0.05, two-tailed Student’s t test. (B) top1α-10 and upf1-1 produce slender seeds as indicated by the length/width ratio. P values were determined by two-tailed Mann-Whitney U-test compared to WT. (C) The peptide of UPF1 identified by IP-MS/MS. (D) Dissected siliques from plants with various genetic backgrounds. Scale bar, 1 mm. (E) qRT-PCR analysis of the expression profiles of TOP1α and UPF1 in adult plants. Relative expression of GOI was normalized against U-BOX expression. Values are mean ± s.d. of three biological replicates. (F) qRT-PCR analysis of the expression profiles of TOP1α and UPF1 during seed development at different days after pollination. Relative expression of GOI was normalized against U-BOX expression. Values are mean ± s.d. of three biological replicates. (G) Seed size of NMD-related mutants. The seed size of upf2-1 is N.A. because of embryonic lethality. P values were determined by two-tailed Mann-Whitney U-test in comparison to wild type. (H) Subcellular localization of TOP1α-GFP (upper panels) and UPF1-mCherry (lower panels) in tobacco leaf epidermal cells. Scale bar, 20 μm. (I) BiFC analysis of the interaction between TOP1α and UPF1 in tobacco leaf epidermal cells. Scale bar, 20 μm. (J) UPF1 subcellular localization shown by cell-fractionation assay. UPF1 protein in nuclear (“N”) or cytoplasmic (“C”) fractions extracted from pistils were detected by anti-FLAG. The nuclear fraction was loaded 10-fold in excess compared to the cytosol fraction. The RUBISCO large subunit (RbcL) stained with Ponceau S and immunoblot analysis using anti-H3 are used as the indicators for cytosol and nuclear fractions, respectively. (K) UPF1 is associated with H3 in vivo as revealed by CoIP. Nuclear protein extracts from the pistils were immunoprecipitated by anti-FLAG. The input and co-immunoprecipitated p [file pbio.3000930.s001.tif]

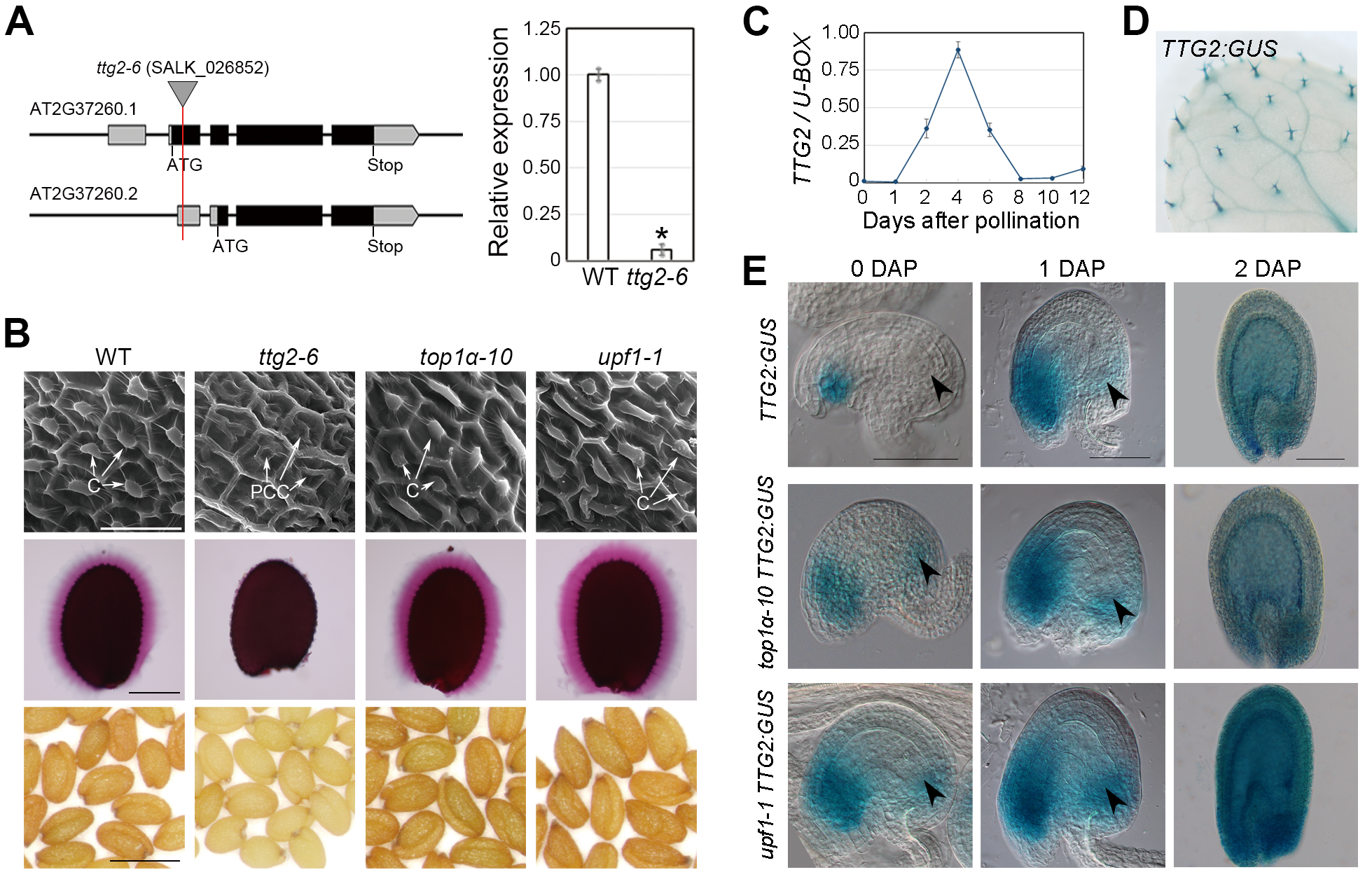

Supplement: S2 Fig — (A) Characterization of ttg2-6. Left panel: Schematic diagram of ttg2-6 insertion site. The coding and untranslated regions are indicated by black and gray boxes, respectively, and introns and other genomic regions are indicated by black lines. Right panel: Relative TTG2 expression in WT and ttg2-6. Values are means ± s.d of three biological replicates. Asterisks indicate significant differences in comparison to wild type. *P < 0.001, two-tailed Student’s t test. (B) Seed coat phenotypes of WT, ttg2-6, top1α-10, and upf1-1. Upper panels: SEM of the seed coat. Scale bar, 50 μm. “C” indicates columella, and “PCC” indicates partially collapsed columella. Middle panels: Seed coat mucilage staining with ruthenium red. Scale bar, 200 μm. Bottom panels: Seed color. Scale bar, 0.5 mm. The data underlying this figure are included in S2 Data. (C) qRT-PCR analysis of the expression profiles of TTG2 during seed development at different DAP. Relative expression of TTG2 was normalized against U-BOX expression. Values are mean ± s.d. of three biological replicates. (D) Typical trichome expression of TTG2:GUS. (E) Representative GUS staining of TTG2:GUS in WT (upper row), top1α-10 (middle row), and upf1-1 (bottom row) backgrounds, and at 0 DAP (left column), 1 DAP (middle column), and 2 DAP (right column). Scale bars, 50 μm (left column), 50 μm (left column), and 200 μm (right column). Arrowheads indicate antipodal cells. DAP, days after pollination; GUS, β-glucuronidase; qRT-PCR, quantitative real-time PCR; SEM, scanning electron microscopy; WT, wild-type plants. (TIF) [file pbio.3000930.s002.tif]

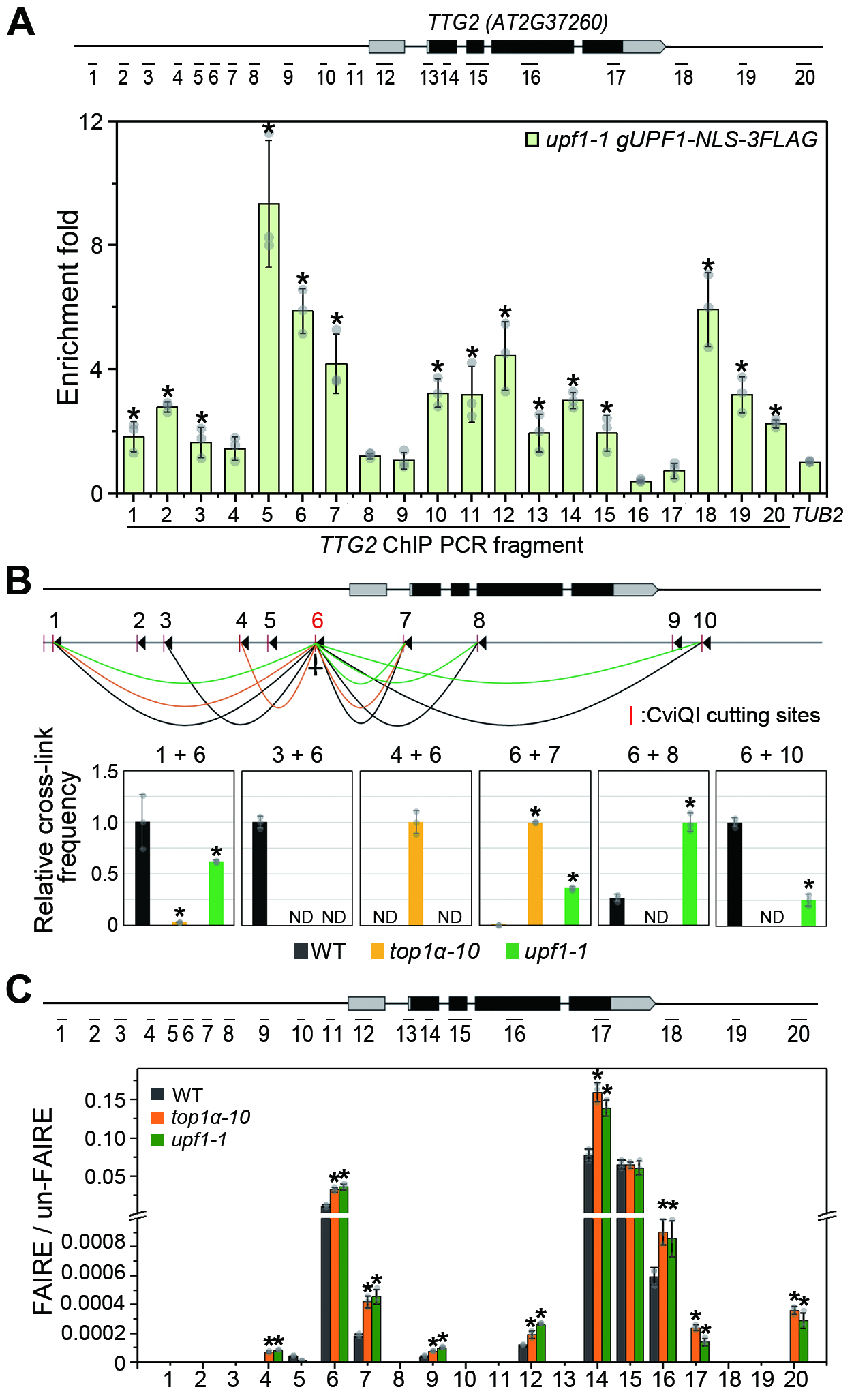

Supplement: S3 Fig — (A) ChIP analysis of UPF1-NLS-3FLAG binding to the TTG2 genomic region. ChIP was performed by anti-FLAG. A TUB2 fragment was amplified as a negative control. Values are mean ± s.d. of three biological replicates. Asterisks indicate significantly high enrichment in comparison to the TUB2 fragment. *P < 0.05, two-tailed Student’s t test. (B) 3C analysis of chromatin looping status at the TTG2 locus. Upper panel: Schematic diagram of the spatial interactions at TTG2 genomic locus. Detectable spatial interactions are linked by black (in wild type), orange (in top1α-10), and green arcs (in upf1-1) between the anchor point (site 6) and surrounding loci. Arrowheads indicate the primers for qPCR. Lower panel: The cross-link frequencies are shown relative to the strongest interaction at each site. Values are mean ± s.d. of three biological replicates. *P < 0.05 as compared to WT, two-tailed Student’s t test. (C) FAIRE analysis of chromatin accessibility of TTG2 locus. Amplicons of cross-linked samples (FAIRE) versus un-cross-linked samples (un-FAIRE) at each site are presented as mean ± s.d. Asterisks indicate significant differences in comparison to wild type. *P < 0.05, two-tailed Student’s t test. The data underlying this figure are included in S2 Data. 3C, chromatin conformation capture; ChIP, chromatin immunoprecipitation; FAIRE, formaldehyde-assisted isolation of regulatory elements; ND, not detectable; qPCR, quantitative PCR; WT, wild-type plants. (TIF) [file pbio.3000930.s003.tif]

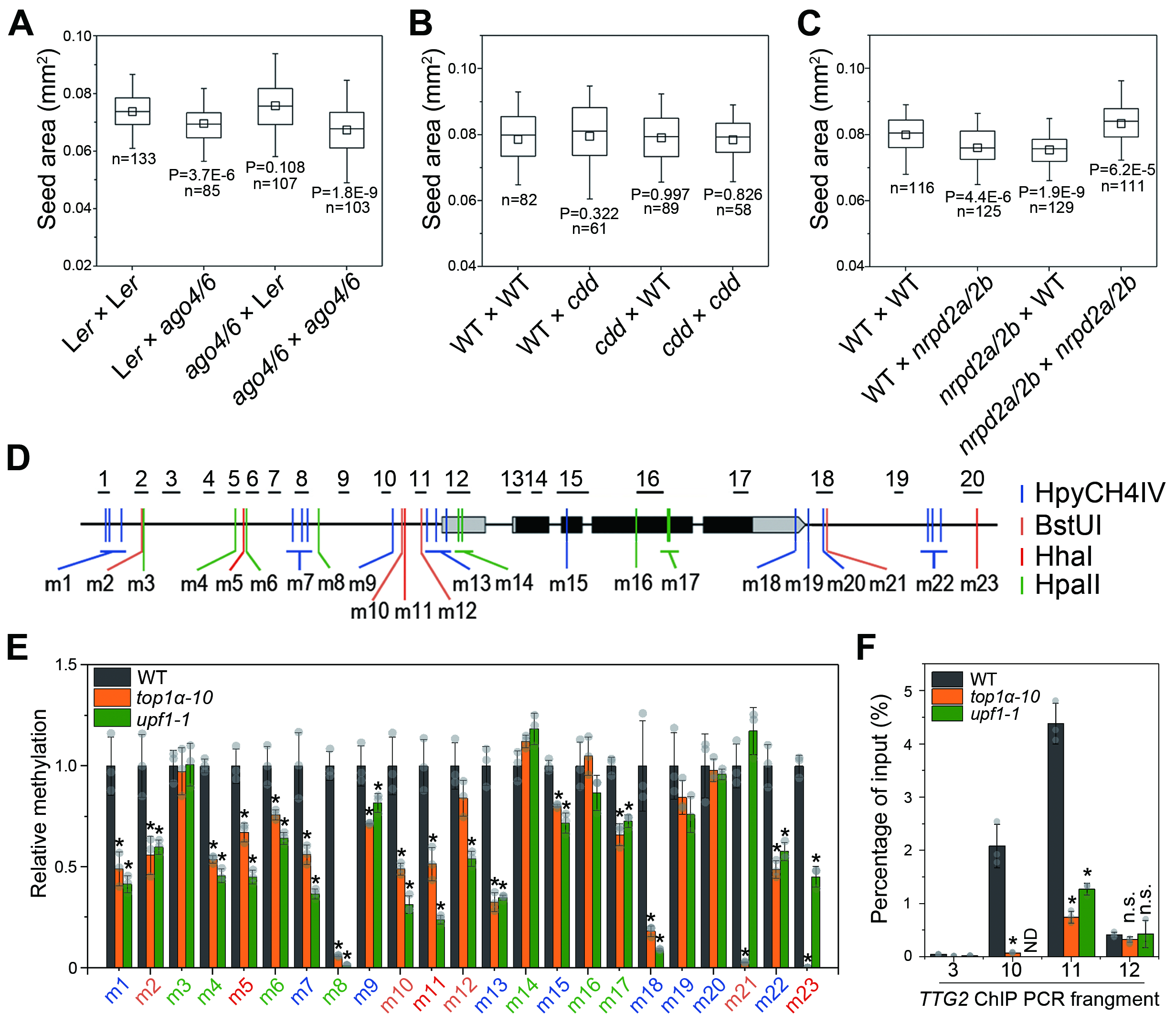

Supplement: S4 Fig — (A-C) Reciprocal crosses of ago4 ago6 (ago4/6) (A), cmt3 drm1 drm2 (cdd) (B), and nrpd2a-2 nrpd2b-1 (nrpd2a/2b) (C) with WT plants. P values were determined by two-tailed Mann-Whitney U-test. (D) The recognition map of selected CpG-sensitive restriction enzymes at the TTG2 locus. The cut sites were indicated in blue (HpyCH4IV), orange (BstUI), red (HhaI), and green (HpaII) strings. The regions to be tested in quantitative real-time PCR are marked as m1 to m23 with the color code of restriction enzymes that digest the corresponding region. PCR fragments of ChIP analysis are aligned above the map. (E) Relative methylation levels at the TTG2 genomic locus. Genomic DNA from pistil was digested by CpG-sensitive restriction enzymes. Undigested genomic DNA was used as an input. The CpG methylation levels were measured by comparing the digested DNA with the input. The relative methylation levels in top1α-10 and upf1-1 backgrounds were normalized against those of WT in each region. Values are mean ± s.d. of three biological replicates. Asterisks indicate significantly low methylation in comparison to WT. *P < 0.05, two-tailed paired Student’s t test. (F) mCIP analysis of the selected regions of TTG2. ChIP PCR fragment 3 and 12 are selected as controls because of no methylation site (fragment 3) or no difference in methylation levels (fragment 12), as indicated in (D and E). ChIP PCR fragment 10 and 11 overlap with region m9–13 as indicated in (D and E). Values are mean ± s.d. of three biological replicates. The asterisks indicate significantly low enrichment compared to WT. *P < 0.05, two-tailed Student’s t test. No statistical difference (n.s), P > 0.05. The data underlying this figure are included in S2 Data. ChIP, chromatin immunoprecipitation; mCIP, methyl-cytosine immunoprecipitation; n, number of seeds examined; ND, not detected; WT, wild-type plants. (TIF) [file pbio.3000930.s004.tif]

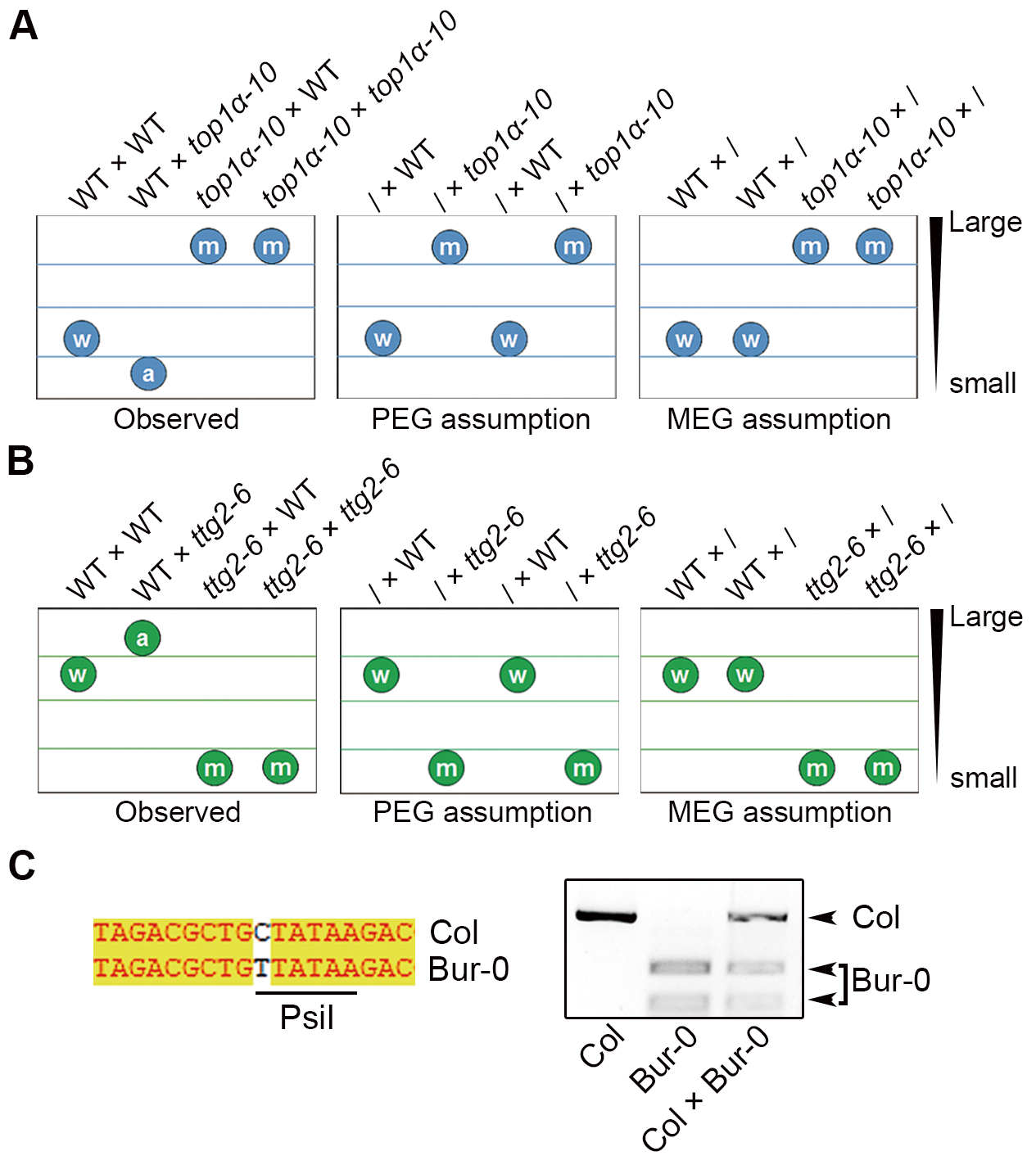

Supplement: S5 Fig — (A and B) The parent-of-origin effect of top1α-10 and ttg2-6 is distinct from that of the mutants of imprinted genes. Schematic diagrams represent the patterns of reciprocal crosses that are related to top1α-10 (A) and ttg2-6 (B). Left panel: Actual observation in this study. Middle panel: Patterns under PEG assumption. Right panel: Patterns under MEG assumption. upf1-1 displays a similar behavior to top1α-10. As the maternal PEG and the paternal MEG are not expressed, the corresponding genotypes are indicated as “/” in the PEG and MEG assumption, respectively. Seed size is marked as WT-like (“w”), mutant-like (“m”), or additional type (“a”). (C) TTG2 is not an imprinted gene. Left panel: The SNP in the coding region of TTG2Bur-0 was used to develop the CAPS marker. Restriction enzyme PsiI digests Bur-0 amplicon, but not Col amplicon. Right panel: CAPS test on cDNA derived from the mRNA of F1 siliques at 2 DAP. CAPS, cleaved amplified polymorphic sequences; DAP, days after pollination; MEG, maternally expressed imprinted gene; PEG, paternally expressed imprinted gene; WT, wild-type plants. (TIF) [file pbio.3000930.s005.tif]

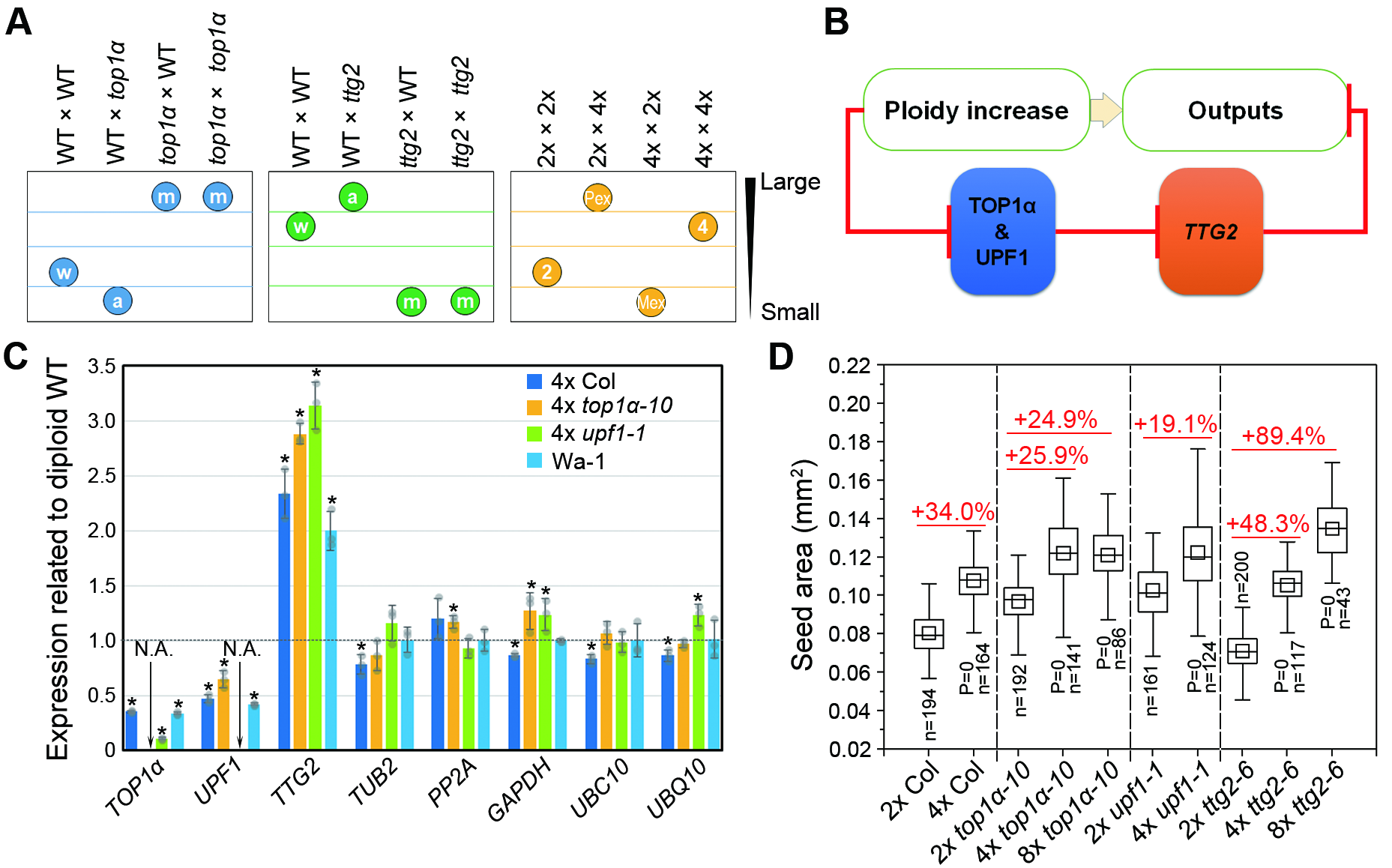

Supplement: S6 Fig — (A) Schematic diagrams that summarize the behaviors in reciprocal crosses. top1α-10 (left panel) and ttg2-6 (middle panel) displayed distinctive phenotypes compared to tetraploids (right panel). upf1-1 displays a similar phenotype to top1α-10. Seed size of reciprocal crosses is marked as “w” (WT-like), “m” (mutant-like) or “a” (additional type). Seed size of interploidy crosses is marked as “Pex” (paternal excess), “4” (tetraploid), “2” (diploid), or “Mex” (maternal excess). (B) TOP1α, UPF1, and TTG2 may compose a feedback console in ploidy increase response. Loss of TOP1α or UPF1 mimics a genome-dosage decrease, whereas loss of TTG2 mimics a genome-dosage increase. (C) Relative gene expression in pistils in tetraploids compared to diploid WT. The relative expression of TOP1α, UPF1, TTG2, and commonly used control genes are presented. Expression values normalized against U-BOX are shown relative to the expression levels in diploid WT. Values are mean ± s.d. of three biological replicates. Asterisks indicate significant differences in comparison to diploid WT. *P < 0.05, two-tailed Student’s t test. TOP1α expression in 4x top1α-10 and UPF1 expression in 4x upf1-1 were not tested (N.A.). (D) Comparison of seed size among diploids, tetraploids, and octoploids in different genetic backgrounds. The percentages of size increase are based on the means of the seed area. P values were determined by two-tailed Mann-Whitney U-test in comparison to corresponding diploids. The data underlying this figure are included in S2 Data. n, number of seeds examined; WT, wild-type plants. (TIF) [file pbio.3000930.s006.tif]

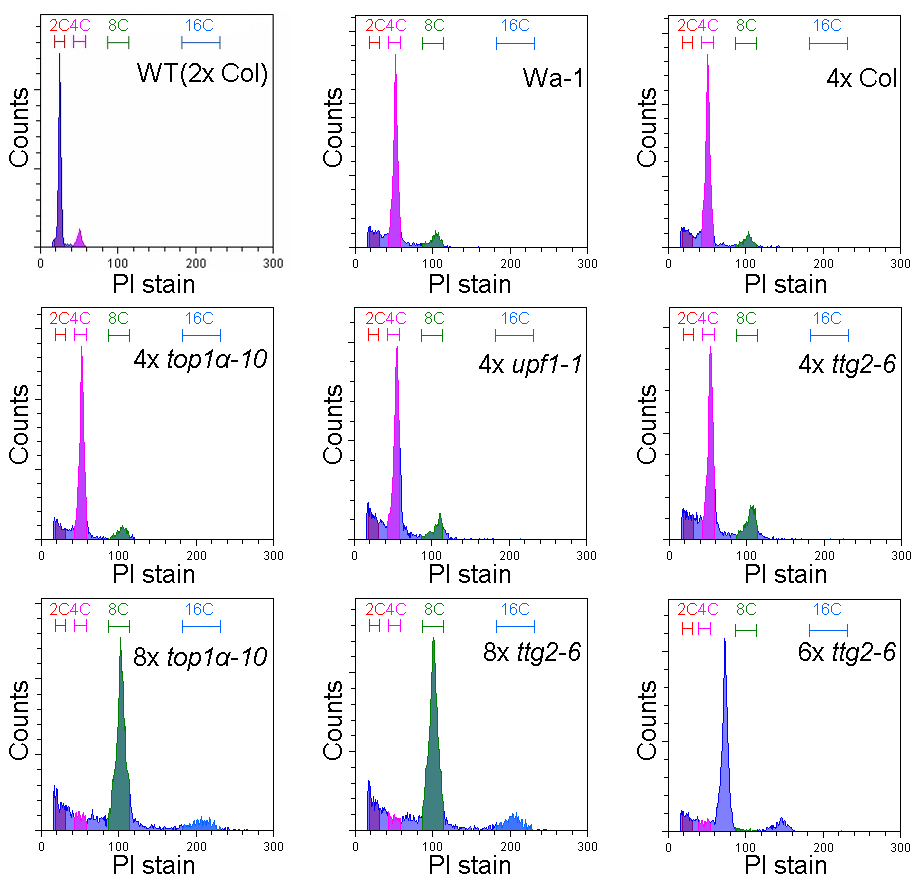

Supplement: S7 Fig — Histograms of 5,000 events are shown with the nuclei in different ploidy indicated as 2C, 4C, 8C, and 16C according to DNA contents. Wild-type plants (Col-0) and Wa-1 (an autotetraploidy accession) are used as controls to calibrate the peaks of 2C, 4C, 8C, and 16C nuclei. Pollinating 8x ttg2-6 with 4x ttg2-6 generated 6x ttg2-6. This is used to show that the peaks are accurate and sensitive enough for determining the ploidy levels. Stage 12 flower buds were used for flow cytometry analysis. Wa-1, Warschau. (TIF) [file pbio.3000930.s007.tif]

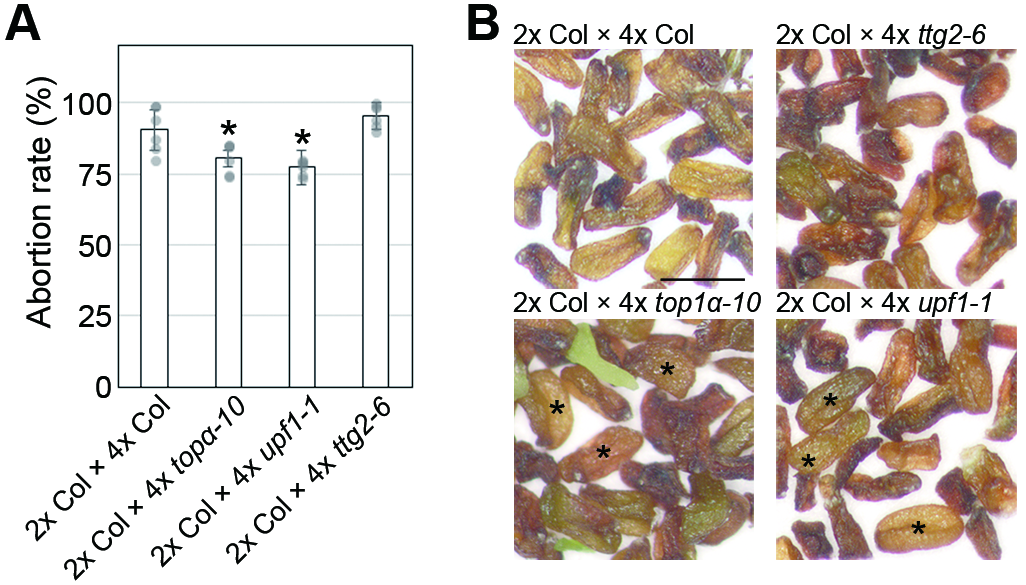

Supplement: S8 Fig — (A) Paternal mutations affect the abortion rate of paternal excess. Values are mean ± s.d. *P < 0.05 as compared to 2x Col × 4x Col, two-tailed Student’s t test. (B) Morphology of the seeds produced by paternal excess with 4x mutants as male parents. Asterisks indicate viable seeds. Scale bar, 0.5 mm. The data underlying this figure are included in S2 Data. (TIF) [file pbio.3000930.s008.tif]

Fig. 1C

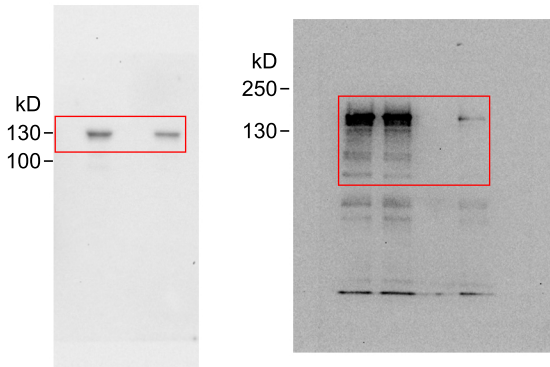

S1J Fig

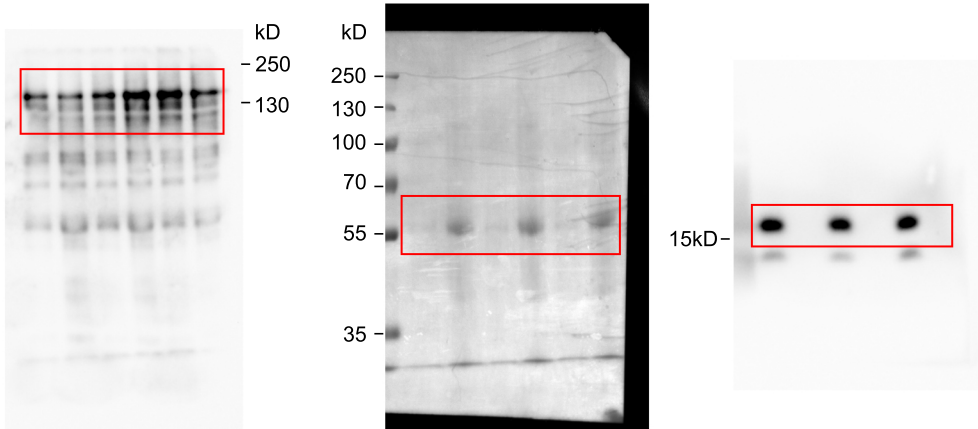

S1K Fig

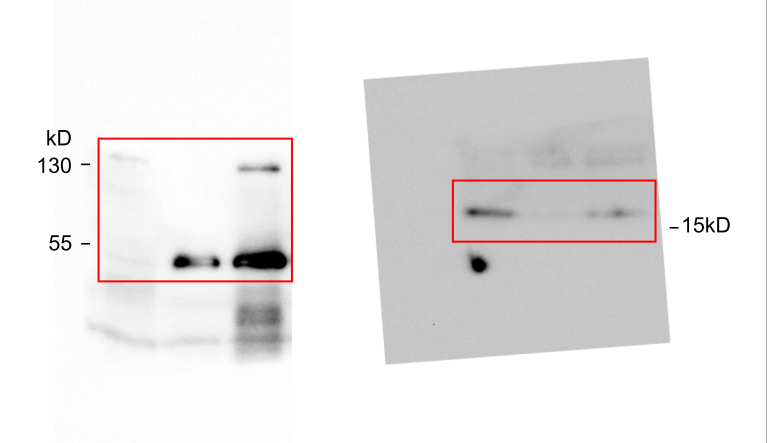

S1L Fig

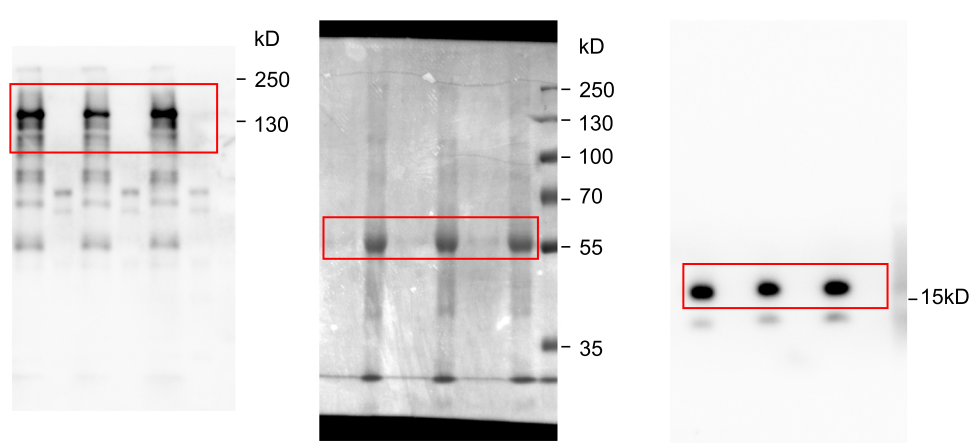

Supplement: S1 Raw Images — From Fig 1C and S1J–S1L Fig. (PDF) [file pbio.3000930.s013.pdf]
