# Supplementary material for: TOP1α, UPF1, and TTG2 regulate seed size in a parental dosage–dependent manner
Source: PLoS Biol. 2020 Nov 6;18(11):e3000930. doi: 10.1371/journal.pbio.3000930 (PMC7673560; doi:10.1371/journal.pbio.3000930)
Supplement: S1 Table — (PDF) [file pbio.3000930.s009.pdf]

S1 Table. Summary of the parent-of-origin genotypes and the corresponding parental *TTG2* dosages.

	TTG2 level in			
Cross combinations [a]	Antipodal cells [b]	Sperm [c]	Relative *TTG2* [d]	Seed phenotype
WT x WT	/	/	balance	Normal
WT x *top1α-10*	/	↑	A<S	Small
top1α-10 x WT	↑↑↑	/	A>S	Large
top1α-10 x *top1α-10*	↑↑↑	↑	A>S	Large
WT x *ttg2-6*	/	↓	A>S	Large
ttg2-6 x WT	↓↓↓	/	A<S	Small
ttg2-6 x *ttg2-6*	↓↓↓	↓	A<S	Small

[a]: Since *UPF1* acts similarly as *TOP1α*, only the phenotypes of *TOP1α* and *TTG2* are listed.

[b]: Maternal *TTG2* is contributed by three antipodal cells, and its upregulation, downregulation and unchanged status are indicated as "↑↑↑", "↓↓↓" and "/", respectively.

[c]: Paternal *TTG2* is contributed by the sperm cell, and its upregulation, downregulation and unchanged status are indicated as "↑", "↓" and "/", respectively.

[d]: "A" indicates relative *TTG2* level in antipodal cells, while "S" indicates relative *TTG2* level in sperms.
