# Supplementary material for: TOP1α, UPF1, and TTG2 regulate seed size in a parental dosage–dependent manner
Source: PLoS Biol. 2020 Nov 6;18(11):e3000930. doi: 10.1371/journal.pbio.3000930 (PMC7673560; doi:10.1371/journal.pbio.3000930)
Supplement: S2 Table — (PDF) [file pbio.3000930.s010.pdf]

S2 Table. Primers used in this study.

1. Primers for UPF1, TOP1 and TTG2 cloning

nYFP fusion

106-UPF1-F	NCGGGATCCATGGATTCTCAACAGAGCGATCTCTTTG
106-UPF1-R	NCCTGTCGACTCAGCCATTGTAAGGATGTTTTGGGTTTTG

cYFP fusion

104-TOP1-F	CATGGTACCATGGGCACTGAAACAGTTTC
104-TOP1-R	GCATCTAGAACGGCGCGAGAATCTGTACTC

mCherry fusion

UPF1-mc-F	NCGGGATCCATGGATTCTCAACAGAGCGATCTCTTTG
UPF1-mc-R	NCCTGTCGACGCCATTGTAAGGATGTTTTGGGTTTTG

GFP fusion

TOP1-GFP-F	CATGGTACCATGGGCACTGAAACAGTTTC
TOP1-GFP-R	GCATCTAGAACGGCGCGAGAATCTGTACTC

BD fusion

BD-UPF1-F	ATTGGCCATGGAGGCCATGGATTCTCAACAGAGCGATCTCTTTG
BD-UPF1-R	TCCCCCGGGTCAGCCATTGTAAGGATGTTTTGGGTTTTG
BD-UPF1-P1-R	TCCCCCGGGTGGGAAGACGAAGTATGCA
BD-UPF1-P2-F	ATTGGCCATGGAGGCCAAGGAGGAAAATGAATTGCG
BD-UPF1-P2-R	TCCCCCGGGTCCAATCATGCCAGCTCCACCAC
BD-UPF1-P3-F	ATTGGCCATGGAGGCCAATGATAATTTTGGTTCGGGTAAC

UPF1 genomic fragment

gUPF1-5'-F	GCTATGACCATGATTACGTGGTTGTTTTTCGTTTATTCTTTTC
gUPF1-5'-R	ATCCAAGGGCGAATTGGTCGCCATTGTAAGGATGTTTTGGGT
UPF1-3EX-F	ATGACAAATGATCCACTGGGTGAGAAAGACAAAACTTGG
UPF1-3EX-R	TACGAACGAAAGCTCGAACTAATATGAGTTTAACACGACCTCC

Add NLS

add-nls-F	AATTGTTGTTGGTAGGGTCAGGCGGAGGAGGTCCGAAAAAGAAGAAACGAA AGGTTGAAGATTGTCCCGCCGGATATAAGA
add-nls-R	AAATCCACCCGGTGAGGGGTCCGGTTCGGCGACCGGAGCACTCGTCAACCA TTTTCCGCTTCTTATATCCGGCGGGACAATCTT

UPF1 promoter

ProUPF1-5'-F	GCTATGACCATGATTACGTGGTTGTTTTTCGTTTATTCTTTTC
ProUPF1-5'-R	TGATCCAAGGGCGAATTGTAGAAAAATCAGGGGAGAAA
GUS-for-5'ProUPF1-F	TTGGATCAGAATTTGCCATGTTACGTCCTGTAGAAACCCCA
GUS-for-5'ProUPF1-R	TCGTCCTTGTAGTCCATGGTTGTTTGCCTCCCTGCTGCGG

UPF1-3EX-F	ATGACAAATGATCCACTGGGTGAGAAAGACAAAACTTGG
UPF1-3EX-R	TACGAACGAAAGCTCGAACTAATATGAGTTTAACACGACCTCC

TOP1 promoter

ProTOP1-F	GCTATGACCATGATTACGCCAAGCTTTAGTGACTTATGGTATTCGCTTTG
ProTOP1-R	CGGGGATCCTCTAGAGTCGACTTCCCGAAAGAACAACG

TTG2 promoter

ProTTG2-F	TGACCATGATTACGCCTTCAGTGGGTGTTTTTGGGATGT
ProTTG2-R	CCGGGGATCCTCTAGACCATTTCCAATACCTTTTACTTCAGC

Primers for *in situ* probes

Probe-top1a-F	TTCCTCCAACAGTAACACATCATCTG
Probe-top1a-R	ACGTGGAATATTTTGAGTCCTTCGT
Probe-UPF1-F	TCTCTTTGACACCGCATCGCAG
Probe-UPF1-R	GTCCCACTTTCGCACCATTCCC

2. Primers for ChIP, DRIP, FAIRE and DNA methylation analysis

For ChIP, DRIP, FAIRE and mCIP

C-TTG2-1-F	CGATTTTCTCATACCGTTGGTT
C-TTG2-1-R	CGTGGCATGTGACTTTTTCTCT
C-TTG2-2-F	GTTGGAAAAGGAACTTACGCTA
C-TTG2-2-R	TGAGAGAGAGGTCCAGAAGATG
C-TTG2-3-F	TGAGCTAAGTCCATTTGAAAGTG
C-TTG2-3-R	GATAGCTGCATCAAGTAACAAGAG
C-TTG2-4-F	AATGTGGCAAGGGTTTTAATGTA
C-TTG2-4-R	GATGTGGAGGGAGAAGTGGAT
C-TTG2-5-F	CTTGACGATTACGAGGAGAAAC
C-TTG2-5-R	AACACAAAATTGATTACGCATATG
C-TTG2-6-F	GTATCAATACTTCTACACCACTTGTTATGC
C-TTG2-6-R	AGCCGCAATATCATCATTCATCA
C-TTG2-7-F	CAGTAAAGTAAAACAGCCGCA
C-TTG2-7-R	TTCCATGATGTAAGGAACGATT
C-TTG2-8-F	GTCTCTGAATTTTTTTTTGGTTTT
C-TTG2-8-R	TGTCACTGTGATTTAGATTGCTATG
C-TTG2-9-F	GTAGAGAGGAGAGTTGATAAGAAAAG
C-TTG2-9-R	GTTGAAGCTTAATCAATGTATATAAAC
C-TTG2-10-F	TAGTGAAAAGTATTGGACAAGATGAC
C-TTG2-10-R	GGTTTATTTCGGTTACACCTCTGT
C-TTG2-11-F	ATAAGTTCTTTTGGTCGCTGTAGT
C-TTG2-11-R	GTTGAAGTTGAAAGTTAGTTGGAA
C-TTG2-12-F	CGTGTATCCATCACAGTGTCGGC
C-TTG2-12-R	CACGACGGTGAATGCAAAAACT

C-TTG2-13-F	ATTTTGCTCCATCTTTCTTTTCCT
C-TTG2-13-R	CACCATCATTCACCTCCATTACTT
C-TTG2-14-F	TACCGTATCTCCGCAAACGACTT
C-TTG2-14-R	CTGCTACTGGCTGATTGAATCTCA
C-TTG2-15-F	TCAACTATTTCTGATAATGATAGGCTG
C-TTG2-15-R	TAGGATACTGCTTCGCTTTGTCTC
C-TTG2-16-F	ATCAGTGGAAGGTCAAGTTTCAGAG
C-TTG2-16-R	CCTTCACTCATCTTCTCTGTTCTGTTT
C-TTG2-17-F	AGTGCTAATTGCAGAGCAAGGAAA
C-TTG2-17-R	GGAAGAGTAGAGGAAGATGGAGGG
C-TTG2-18-F	AGTAACCACGTTGGTATTGTACTAC
C-TTG2-18-R	GATCTTGACATGTGATTCTTTCTC
C-TTG2-19-F	TTAGCAACTCTCTCAATCTCATCT
C-TTG2-19-R	TAATAATAGCATCTTCTCTTGTCTTG
C-TTG2-20-F	TCCTGACAGCCATCAACTTCTT
C-TTG2-20-R	CGCAACTTTTGTTCCCCATACT

For 3C

3C-TTG2-CviQI-P1	TCTGGAAGCAACAATGGCGAT
3C-TTG2-CviQI-P2	CACTGTTAGAAATGATAATGGAGATTG
3C-TTG2-CviQI-P3	GGATGCCTCCAGGTAAGTCAA
3C-TTG2-CviQI-P4	AGCGTCAAAAACCAACAACAT
3C-TTG2-CviQI-P5	AATTGTCTAACTAATATGTTAGGTTAGTTAAG
3C-TTG2-CviQI-P6	AAGACTACAGCGACCAAAAGAACT
3C-TTG2-CviQI-P7	AGATGGAGCAAAATCAAAGAAGA
3C-TTG2-CviQI-P8	CCAGTAGATGTCTCTGATTCCG
3C-TTG2-CviQI-P9	CGAAAATGTAAACAAGTATCGGATAG
3C-TTG2-CviQI-P10	TCTAAGGACACAACCAATGCC
3C-TTG2-TaqI-P1	CGAAAGTGAGATTTGACCGAA
3C-TTG2-TaqI-P2	TTTTCTTTCCCATTTTCTGTTACT
3C-TTG2-TaqI-P3	CAGTGGATTGTCTTTTGGTTCAG
3C-TTG2-TaqI-P4	GAAATGCTGAACGGGACTTGG
3C-TTG2-TaqI-P5	GGTGTAACCGAAATAAACCGAAT
3C-TTG2-TaqI-P6	ACGACTTGCCACGAGATTGTAG
3C-TTG2-TaqI-P7	TGTCTCCAAAGCAACCGTCTC
3C-TTG2-TaqI-P8	ACTCAAAGCCATCTTGTCCTCT
3C-TTG2-TaqI-P9	TATTTTTTAGGGGTAACTTTCTTCAA
3C-TTG2-TaqI-P10	ATATAACTACCATGATTTATAGCACTTTAA
3C-TTG2-TaqI-P11	CACAGAGTATCGTCCAGCAGTAT

For methylation map

m1-F	CGAAAGTGAGATTTGACCGAA
m1-R	TCTGGAAGCAACAATGGCGAT
m2-F	CGATTTTCTCATACCGTTGGTT

m2-R	TGAGAGAGAGGTCCAGAAGATG
m3-F	GTTGGAAAAGGAACTTACGCTA
m3-R	TGAGAGAGAGGTCCAGAAGATG
m4-F	CTTGACGATTACGAGGAGAAAC
m4-R	AACACAAAATTGATTACGCATATG
m5-F	CTTGACGATTACGAGGAGAAAC
m5-R	AGCCGCAATATCATCATTCATCA
m6-F	TTCACCTCAATAGAAATAATCATACACAC
m6-R	AGCCGCAATATCATCATTCATCA
m7-F	TACTTGCTCTGTTATTTGTTGTGAT
m7-R	AGCGTCAAAAACCAACAACAT
m8-F	GAAATGCTGAACGGGACTTGG
m8-R	GTTGAAGCTTAATCAATGTATATAAAC
m9-F	TAGTGAAAAGTATTGGACAAGATGAC
m9-R	AAGACTACAGCGACCAAAAGAACT
m10-F	GGTGTAACCGAAATAAACCGAAT
m10-R	AAGACTACAGCGACCAAAAGAACT
m11-F	GGTGTAACCGAAATAAACCGAAT
m11-R	AAGACTACAGCGACCAAAAGAACT
m12-F	ATAAGTTCTTTTGGTCGCTGTAGT
m12-R	GTTGAAGTTGAAAGTTAGTTGGAA
m13-F	ATAAGTTCTTTTGGTCGCTGTAGT
m13-R	CACGACGGTGAATGCAAAAACT
m14-F	CGTGTATCCATCACAGTGTCGGC
m14-R	CACGACGGTGAATGCAAAAACT
m15-F	TCAACTATTTCTGATAATGATAGGCTG
m15-R	TAGGATACTGCTTCGCTTTGTCTC
m16-F	ATGGATACAACTGGAGGAAATACGG
m16-R	CCTTCACTCATCTTCTCTGTTCTGTTT
m17-F	TTATTACACCATTTGAGTTTGCTG
m17-R	CTTCTCGTTCTTCCTGTTATCAAA
m18-F	CAATCACCATTTGCTCTTCACTC
m18-R	GAAGAAAGTTACCCCTAAAAAATA
m19-F	TATTTTTTAGGGGTAACTTTCTTCAA
m19-R	TTGAGTTACACCGATGGAAATTG
m20-F	AGTAACCACGTTGGTATTGTACTAC
m20-R	GATCTTGACATGTGATTCTTTCTC
m21-F	AGTAACCACGTTGGTATTGTACTAC
m21-R	GATCTTGACATGTGATTCTTTCTC
m22-F	TTAGCAACTCTCTCAATCTCATCT
m22-R	CACAGAGTATCGTCCAGCAGTAT
m23-F	TCCTGACAGCCATCAACTTCTT
m23-R	CGCAACTTTTGTTCCCCATACT

3. Primers for qRT-PCR

qRT-TTG2-F	AGTGCTAATTGCAGAGCAAGG
qRT-TTG2-R	GAAGAGTAGAGGAAGATGGAGGG
qRT-UPF1-F	ATCCCAGGATGATGGACAGCAG
qRT-UPF1-R	CGAATTTGGGAGACCCTGAGAC
qRT-U-BOX-F	TCTTCTTCTGCTACATCTACTCTC
qRT-U-BOX-R	AGTGTGTGAACCCGTGAAC
qRT-PP2A-F	TTGGTGCTCAGATGAGGGAGAG
qRT-PP2A-R	TTCACCAGCTGAAAGTCGCTTAG
qRT-GAPDH-F	ACCACTGTCCACTCTATCACTGC
qRT-GAPDH-R	TGAGGGATGGCAACACTTTCCC
qRT-ACT2-F	TCTTCCGCTCTTTCTTTCCAAGC
qRT-ACT2-R	ACCATTGTCACACACGATTGGTTG
qRT-UBC10-F	CCATCCCAACATTAACAGCAACGG
qRT-UBC10-R	ACAGATCGATAGCAGCACCTTGG
qRT-TUB2-F	TAACAACTGGGCCAAGGGACAC
qRT-TUB2-R	ACAAACCTGGAACCCTTGGAGAC
qRT-UBQ10-F	TAAGACGTTGACTGGGAAAACTA
qRT-UBQ10-R	AGTTCTTCCATCTTCAAGTTGC
